# TRIM28 multi-domain protein regulates cancer stem cell population in breast tumor development

**DOI:** 10.18632/oncotarget.13273

**Published:** 2016-11-10

**Authors:** Patrycja Czerwińska, Parantu K. Shah, Katarzyna Tomczak, Marta Klimczak, Sylwia Mazurek, Barbara Sozańska, Przemysław Biecek, Konstanty Korski, Violetta Filas, Andrzej Mackiewicz, Jannik N. Andersen, Maciej Wiznerowicz

**Affiliations:** ^1^ Laboratory for Gene Therapy, Department of Diagnostics and Cancer Immunology, Greater Poland Cancer Centre, Poznan, Poland; ^2^ Department of Cancer Immunology, Chair of Medical Biotechnology, Poznan University of Medical Sciences, Poznan, Poland; ^3^ Postgraduate School of Molecular Medicine, Medical University of Warsaw, Warsaw, Poland; ^4^ Institute for Applied Cancer Science, University of Texas MD Anderson Cancer Center, Houston, Texas, USA; ^5^ Faculty of Mathematics and Information Science, Warsaw University of Technology, Warsaw, Poland; ^6^ Faculty of Mathematics, Informatics, and Mechanics, University of Warsaw, Warsaw, Poland; ^7^ Department of Cancer Pathology, Greater Poland Cancer Centre, Poznan, Poland

**Keywords:** TRIM28, KAP1, breast cancer stem cells, pluripotency, epigenetics

## Abstract

The expression of Tripartite motif-containing protein 28 (TRIM28)/Krüppel-associated box (KRAB)-associated protein 1 (KAP1), is elevated in at least 14 tumor types, including solid and hematopoietic tumors. High level of *TRIM28* is associated with triple-negative subtype of breast cancer (TNBC), which shows higher aggressiveness and lower survival rates. Interestingly, TRIM28 is essential for maintaining the pluripotent phenotype in embryonic stem cells. Following on that finding, we evaluated the role of TRIM28 protein in the regulation of breast cancer stem cells (CSC) populations and tumorigenesis *in vitro* and *in vivo*. Downregulation of *TRIM28* expression in xenografts led to deceased expression of pluripotency and mesenchymal markers, as well as inhibition of signaling pathways involved in the complex mechanism of CSC maintenance. Moreover, *TRIM28* depletion reduced the ability of cancer cells to induce tumor growth when subcutaneously injected in limiting dilutions. Our data demonstrate that the downregulation of *TRIM28* gene expression reduced the ability of CSCs to self-renew that resulted in significant reduction of tumor growth. Loss of function of *TRIM28* leads to dysregulation of cell cycle, cellular response to stress, cancer cell metabolism, and inhibition of oxidative phosphorylation. All these mechanisms directly regulate maintenance of CSC population. Our original results revealed the role of the TRIM28 in regulating the CSC population in breast cancer. These findings may pave the way to novel and more effective therapies targeting cancer stem cells in breast tumors.

## INTRODUCTION

Overcoming resistance to chemotherapy and radiotherapy in solid tumors is one of the fundamental issues of clinical oncology. Considerable responsibility for resistance to conventional treatments, as well as the processes of metastasis and relapse, has been attributed to the existence of cancer stem cells (CSCs) [1]. These cells, also known as tumor-initiating cells (TICs) are rare within the tumor and exhibit stem cell properties such as the capacity of self-renewal, pluripotency, highly tumorigenic potential and resistance to therapies. Maintenance of normal stem cells as well as cancer stem cells is controlled by master transcription factors that regulate the expression of stem cell-specific genes. The core transcription machinery form multiple regulatory connections with other transcription factors, epigenetic regulators and non-coding RNAs, developmental signaling pathways, and other modifiers, that, together, contribute to the self-renewal and pluripotency of stem cells and, similarly, cancer stem cells [2–4]. This internal regulatory network is sustained by environmental cues from the stem cell niche [5–7]. Recently, it was demonstrated that pluripotency control is hardwired to the cell-cycle machinery. S and G2 phase-associated pathways were demonstrated to trigger selective preference toward pluripotency maintenance when the progression of stem cell through the cell cycle was perturbed [8]. Moreover, pluripotency of stem cells is tightly associated with their metabolism [9]. The metabolic profile of stem cells was found to be different from that of their terminally differentiated somatic counterparts and shifted from oxidative phosphorylation to aerobic glycolysis [9]. In contrast, cancer stem cells are less glycolytic and more dependent on mitochondrial respiration. As presented by Viale *et al* [10], cancer stem cells isolated from pancreatic tumor spheres expressed higher level of genes involved in several metabolic pathways (i.e. mitochondrial electron transport chain (ETC), lysosome activity, autophagy, mitochondrial and peroxisomal β-oxidation) and suggested that cancer stem cells have increased mitochondrial activity. All these biological processes keep the cancer cells in the pluripotent state. However, the exact molecular targets that regulate these molecular processes remain largely unknown.

Tripartite motif-containing protein 28 (TRIM28) is thought to regulate the dynamic organization of chromatin structure by influencing epigenetic patterns and chromatin compaction and may thus play an important role in the homeostasis of cancer cells. TRIM28, also known as transcription intermediary factor 1 (TIF1β) or Krüppel-associated box (KRAB)-associated protein 1 (KAP1), is a universal co-repressor for a family of KRAB domain-containing zinc finger proteins (KRAB-ZFPs), which constitute the single largest group of transcriptional repressors encoded by the genomes of higher organisms [11].

TRIM28 is essential for maintaining the stem cell phenotype of the induced pluripotent stem cells and the embryonic stem cells (ESC). Mouse embryos deficient in *Trim28* die before gastrulation, suggesting that Trim28 plays a pivotal role in the self-renewal of ESC [12, 13]. Recent studies have indicated importance of KRAB/TRIM28-mediated epigenetic regulation in both B-lymphocyte and T-lymphocyte differentiation and homeostasis [14]. Furthermore, TRIM28 has been reported to regulate apoptosis in a manner independent of its transcriptional activities. By recruiting histone deacetylase 1 (HDAC1) to the MDM2-p53 complex, TRIM28 acts cooperatively with MDM2 to induce p53 degradation [15, 16]. This effect suggests that TRIM28 may promote neoplastic transformation by suppressing apoptosis. Moreover, TRIM28 has been implicated in the DNA-damage response (DDR) pathway [17]. Additionally, TRIM28 is involved in the fibroblast-specific protein 1 (FSP-1)-mediated epithelial to mesenchymal transition (EMT), which is considered to be an important mechanism for the acquisition of metastatic properties [18]. Recent studies have demonstrated the role of TRIM28 protein in autophagy, a stress-induced process that has been suggested to maintain the CD44^+^/CD24^−/low^ breast cancer stem-like phenotype [19–21].

Increased levels of TRIM28 protein have been observed in liver, gastric, lung, breast, pancreatic and prostate cancer. In patients with gastric or pancreatic cancer, high levels of TRIM28 correlate with a significantly lower survival rate [22–24]. To date, many results have indicated that TRIM28 plays a critical role in the proliferation and differentiation of both normal and tumor cells. Despite many efforts to elucidate the cellular functions and associated molecular mechanisms of TRIM28, the role of this protein in tumorigenesis remains to be elucidated.

Although a considerable number of studies have revealed the roles of TRIM28 protein in experimental systems, little is known about the correlation between *TRIM28* gene expression and clinical outcome in breast tumors. Here, we demonstrated that TRIM28-depletion in breast cancer cells lead to significant reduction of tumor growth *in vivo*. Further analyses have revealed strong reduction of specific markers and activity of molecular pathways that are strongly associated with the breast cancer stem cells. Importantly, direct involvement of CSC in the TRIM28-knockdown phenotype was confirmed by the functional studies. This report demonstrates for the first time the engagement of TRIM28 protein in the regulation of CSCs in breast cancer, which facilitates tumor progression.

## RESULTS

### *TRIM28* gene expression is associated with more aggressive breast cancers

**Figure 1:**
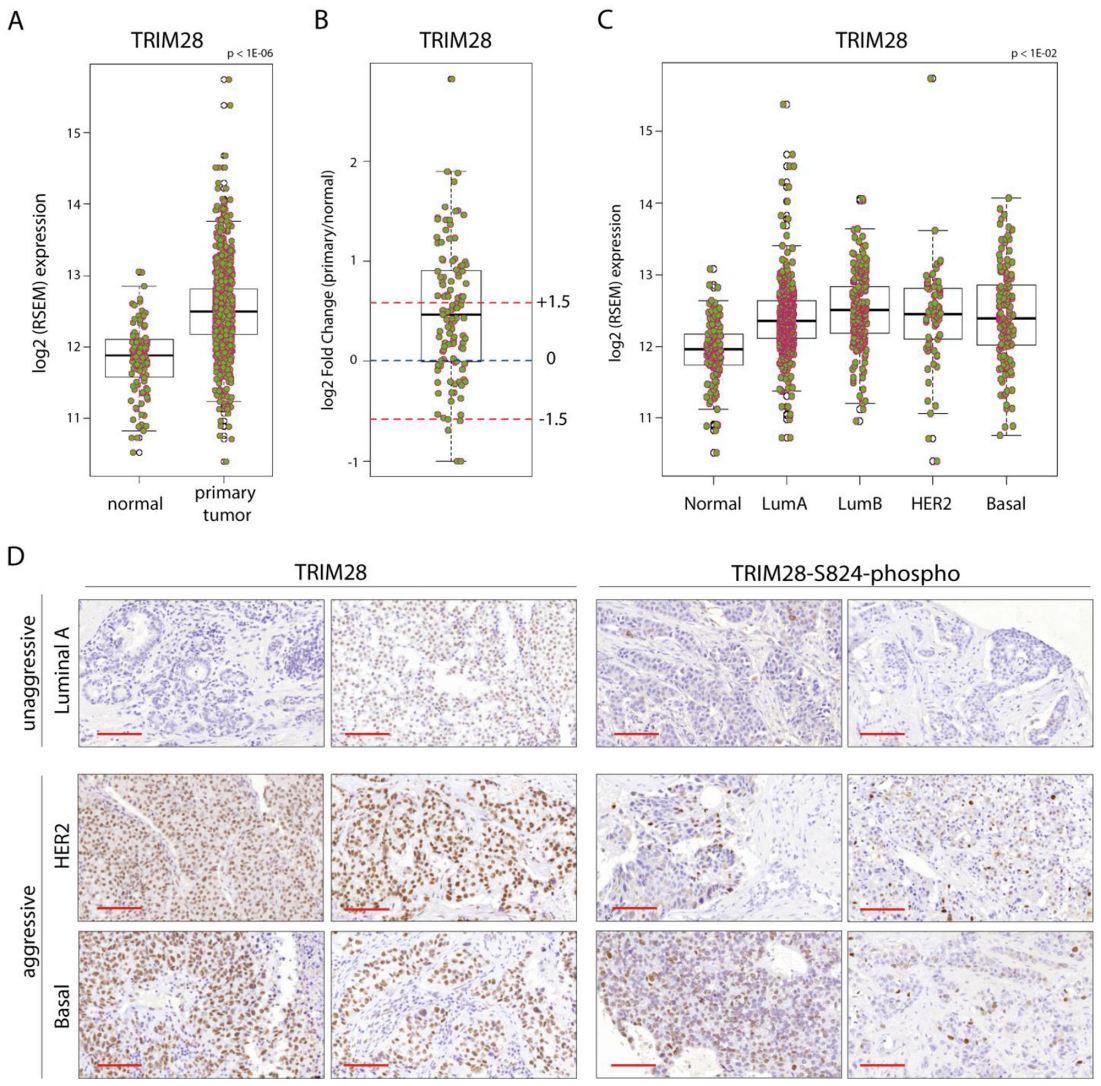
*TRIM28* gene is overexpressed in breast cancer **A.** Box plot presents the *TRIM28* gene expression level in primary breast tumor and normal tissue from TCGA project. The black line presents median, box shows interquantile region and whiskers - the highest (max) and the lowest (min) value. Outliers are also depicted. **B.** Differential expression analysis of TCGA BRCA patient samples for which tumor and matched normal tissues are available. Fold changes +/− 1.5 fold change are depicted. The black line presents median, box shows interquantile region and whiskers - the highest (max) and the lowest (min) value. Outliers are also depicted. **C.** *TRIM28* gene expression is distinct between different BRCA PAM50 intrinsic subtypes. **D.** IHC for total TRIM28 and phosphorylated TRIM28 protein (TRIM28-S824) in selected breast cancer samples confirmed higher levels of TRIM28/phospho-TRIM28 in more aggressive breast cancer subtypes (basal and HER2+). Scale = 100 μm.

Differential expression analysis of different tumor types from the *Oncomine* database suggested that *TRIM28* is differentially expressed in 14 tumor types, including solid and hematopoietic tumors. TRIM28 was in top 10% differentially expressed genes (p < 1E-04; |FC| > 1.5; Gene Rank (%) < 10 %) between cancer and adjacent normal tissue in 33 datasets from the *Oncomine* database (Supplementary Table S1). *TRIM28* is also significantly differentially expressed in the TCGA breast invasive carcinoma (BRCA) gene expression profiles of more than 1000 patients compared with normal tissues (Figure 1A; p < 1E-06). A total of 42% (47/111) of the patients for whom paired gene expression profiles of tumor and matched normal tissues are available showed more than 1.5-fold *TRIM28* overexpression in their tumor tissues (Figure 1B). Moreover, *TRIM28* expression is distinct between different BRCA intrinsic subtypes (p < 0.01), and *TRIM28* high-expressing patients are depleted in the less aggressive luminal A subtype of TCGA BRCA (p = 1.2E-03; Figure 1C). TRIM28 is also associated with triple-negative tumors in TCGA BRCA (Supplementary Figure S1A; p = 2.2E-16) and in the dataset of Stickeler *et al*. [25], (p = 4.2E-06). IHC staining confirms that more aggressive breast cancer subtypes are more frequently positive for TRIM28 and TRIM28-S824-phospho than luminal A subtype (Figure 1D). *TRIM28* was also overexpressed in patients with distant detectable metastasis (TCGA pathology M stage), with those in the M1 stage showing the highest gene expression (Supplementary Figure S1B). Finally, *TRIM28* high-expressing patients had significantly worse overall survival (p = 6E-04) and recurrence-free survival (p = 0.01) than *TRIM28* low-expressing patients (Table 1) in a cohort of treated patients [26]. Therefore, we hypothesized that TRIM28 may play a role in aggressive breast tumor progression with stem cell-like features.

**Table 1:** Summary of survival analysis of breast cancer patients with high and low expression levels of the ***TRIM28*** gene

SURVIVAL TYPE	PATIENT WITH HIGH EXPRESSION	PATIENT WITH LOW EXPRESSION	LOG RANK p-value	HAZARD RATIO
**RECURRENCE FREE**	694	2346	**6.00E-04**	1.23
**OVERALL**	357	758	**0.01**	1.37
**DISTANCE METASTASIS FREE**	337	820	0.12	1.19
**PALLIATIVE PERFORMANCE SCALE**	98	253	0.44	0.89

Survival analysis was carried out using breast cancer datasets available at http://kmplot.com/analysis/ (Gyorffy et al., 2010 Breast Cancer Research and Treatment 123 : 725-731).

### Knockdown of TRIM28 for 24h has little impact on tumorigenic and stem cell like properties of breast cancer cell lines *in vitro*

**Figure 2:**
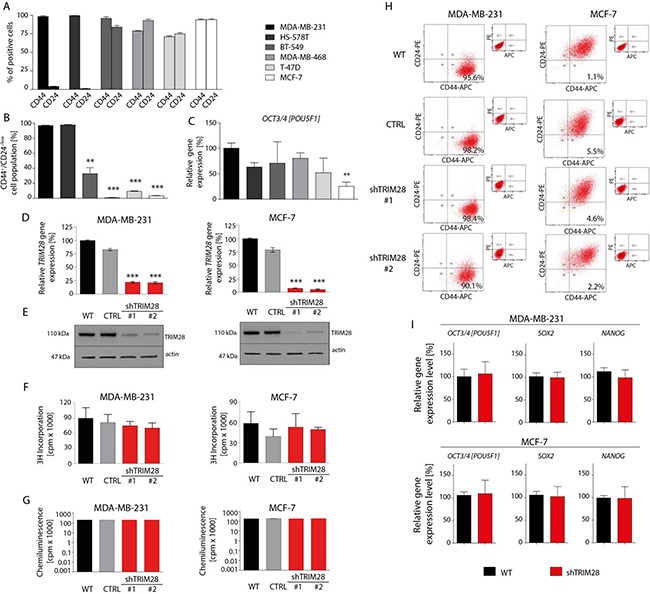
*TRIM28* knockdown does not affect breast cancer cell proliferation, cell viability and the percentage of CD44^+^/CD24^−/low^ breast cancer stem cell population *in vitro* **A.** 6 breast cancer cell lines (MDA-MB-231, HS-578T, BT-549, MDA-MB-468, T-47D and MCF-7) are characterized by different proportion of CD44-positive and CD24-positive cells as determined using FACS analysis. **B.** Identification of a CD44^+^/CD24^−/low^ subpopulation in breast cancer cell lines by flow cytometry. Relatively to MDA-MB-231 breast cancer cell line, MCF-7 and T-47D luminal breast cancer cell line and MDA-MB-468 basal-like breast cancer cell line are poor in the population of CD44^+^/CD24^−/low^ cancer stem cells. Error bars, SD; n = 3; ** p < 0.01; *** p < 0.001. **C.** The level of *OCT3/4* pluripotency marker in selected breast cancer cell lines *in vitro* was determined using RT-qPCR analysis. Relatively to MDA-MB-231 breast cancer cell line, MCF-7 cells express *OCT3/4* at the lowest level *in vitro*. Error bars, SD; n = 4; ** p < 0.01. **D, E.** MDA-MB-231 and MCF-7 cells stably infected with lentiviral vectors expressing *TRIM28* shRNA (sh#1 or sh#2) or with empty vector (CTRL) were analyzed using RT-qPCR (A) and Western blot (B) for TRIM28 gene and protein levels. Error bars, SD; n = 3; *** p < 0.001. **F, G.** *TRIM28* downregulation does not affect cell proliferation (F) and viability (G) *in vitro* as determined using an ^3^H-thymidine-incorporation assay and ATPlite™ Luminescence Assay, respectively. Error bars, SD; n = 4; p > 0.05. **H.** The comparison of CD44^+^/CD24^−/low^ cells frequency in breast cancer cell lines upon reduction of TRIM28 level *in vitro*. *TRIM28* knockdown does not affect the percentage of breast cancer stem cell population *in vitro*. **I.** *TRIM28* downregulation does not affect the expression of pluripotency markers *OCT3/4*, *SOX2* and *NANOG in vitro* in MDA-MB-231 (upper panel) and MCF-7 (bottom panel) breast cancer cell lines as determined using RT-qPCR. Error bars, SD; n = 4; p > 0.05.

To understand association of TRIM28 function with stem cell-like properties in breast cancer, we characterized presence of stemness markers in a panel of breast cancer cell lines that are annotated with Basal B (MDA-MB-231, HS-578T, BT-549), Basal-like (MDA-MB-468) and Luminal (T-47D and MCF-7) like characteristics [27, 28]. Indeed, the examined breast cancer cell lines are characterized by different proportion of CD44 and CD24 expressing cells. These cell surface markers are frequently used for breast cancer stem cell separation (Figure 2A). The MDA-MB-231 and HS-578T cell lines possess the highest number of CD44^+^CD24^−/low^ cells (Figure 2B) while MCF-7 and T-47D luminal as well as MDA-MB-468 basal-like breast cancer cell line are characterized by very low number of CD44^+^CD24^−/low^ cells. Cancer stem cells also express common pluripotency markers such as *OCT3/4* [*POU5F1*] transcription factor. We saw the highest relative expression of OCT3/4 in MDA-MB-231 cells and the lowest relative expression in MCF-7 cell line (Figure 2C). Therefore, we prioritize MDA-MB-231 and MCF-7 for further studies (Figure 2 and Supplementary Figure S2).

We introduced *TRIM28* shRNAs in these cell lines and studied its impact on cell proliferation and viability. Even though the shRNA reduced levels of *TRIM28* endogenous gene by approximately 75% to 85% (Figure 2D and 2E), we did not see any impact of this knockdown on cell proliferation (^3^H-thymidine-incorporation assay; Figure 2F) and viability (ATPlite™ Luminescence Assay; Figure 2G). Similar studies in the extended panel showed the same results (Supplementary Figure S2A-S2D). In accordance to this observation, *TRIM28* reduction did not change the percentage of CSCs characterized by the CD44^+^CD24^−/low^ phenotype *in vitro* in the tested breast cancer cell lines (Figure 2H and Supplementary Figure S2E). Finally, *TRIM28* gene downregulation did not suppress the expression of selected pluripotency markers *in vitro* as determined using RT-qPCR (Figure 2I and Supplementary Figure S2F).

**Figure 3:**
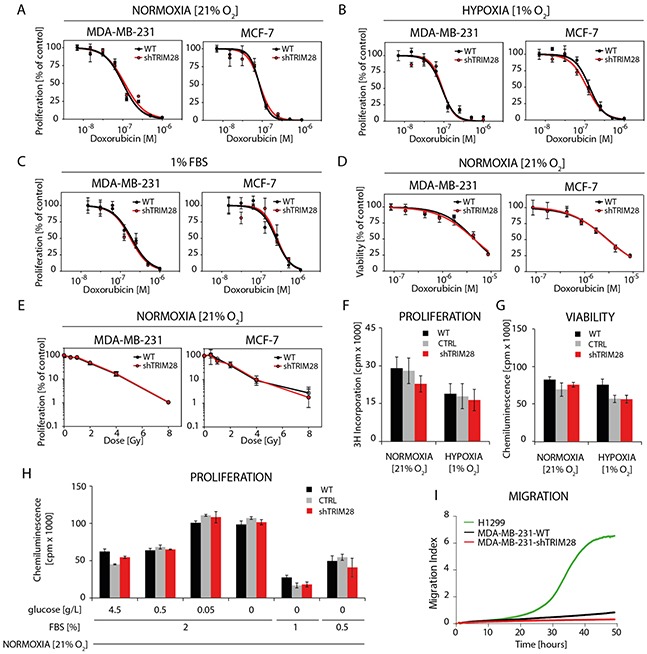
*TRIM28* knockdown does not affect breast cancer cell chemo- and radioresistance in vitro **A, B, C.** The dose response curves show the relative proliferation *in vitro* (^3^H-thymidine incorporation assay) of TRIM28^WT^ and TRIM28^KD^ (sh#1) cells after doxorubicin treatment in normoxia (A), hypoxia (B) and low level of serum (C). Error bars, SD; n = 4; p > 0.05. **D.** The dose response curves presenting relative cell viability *in vitro* (ATPlite™ Luminescence Assay) of TRIM28^WT^ and TRIM28^KD^ (sh#1) cells after doxorubicin treatment in normoxia. Error bars, SD; n = 4; p > 0.05. **E.** Radiation dose response curves show the relative proliferation of TRIM28^WT^ and TRIM28^KD^ (sh#1) cells 80 hours after γ-irradiation. Proliferation was analyzed using an ^3^H-thymidine incorporation assay. Error bars, SD; n = 3; p > 0.05. **F, G, H.** The proliferation and viability of breast cancer cells in normoxia (F), hypoxia (G) or in low level of serum and/or glucose (H) is not affected *in vitro* by *TRIM28* knockdown as determined using ^3^H-thymidine incorporation assay and ATPlite™ Luminescence Assay, respectively. Error bars, SD; n = 3; p > 0.05. **I.** Preliminary results from *in vitro* migration assay using xCELLigence® RTCA DP instrument revealed very low potential of MDA-MB-231 triple-negative breast cancer cells to migrate *in vitro* when attracted with 10% FBS containing medium. Lung cancer H1299 cell line was used as a positive control.

Next, we analyzed the chemo- and radioresistance of selected cell lines (Figure 3A-3E and Supplementary Figure S3). To our surprise, *TRIM28* depletion did not sensitize MDA-MB-231 and MCF-7 cells or other cell lines from the panel to doxorubicin, neither in normoxia (21% O_2_), hypoxia (1% O_2_) nor in serum-restricted conditions (1% FBS) as determined using ^3^H-thymidine-incorporation assay (Figure 3A-3C) or ATPlite™ Luminescence Assay (Figure 3D). Similarly, *TRIM28* knockdown did not sensitize breast cancer cells to irradiation *in vitro* over the dose range of 0-8 Gy (Figure 3E). We further performed series of experiments testing the effect of *TRIM28* reduction in different culture conditions (Figure 3F-3H) and observed, that neither in hypoxia (1% O_2_) nor in lowered concentration of serum (2, 1 or 0.5% of FBS) together with low glucose concentrations (0.5, 0.05 or 0 g/L) *TRIM28* knockdown affect cell proliferation or viability *in vitro*. Due to enhanced invasive properties of CD44^+^/CD24^−/low^ cancer stem cell population [29] we further tested the migration using xCELLigence® RTCA PD instrument. We observed that MDA-MB-231 cells did not migrate *in vitro* in contrast to control lung cancer cell line H1299 and thus, we abandoned further analyses of cell migration/invasion *in vitro*.

Therefore, we conclude that *TRIM28* knockdown does not affect cell homeostasis in breast cancer cell lines *in vitro*.

### TRIM28 protein regulates tumor growth *in vivo*

**Figure 4:**
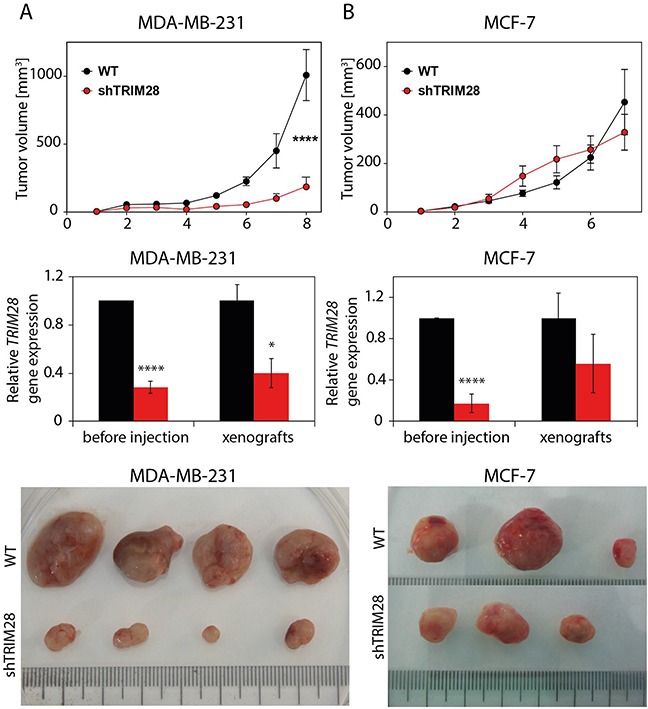
TRIM28 protein regulates tumor growth *in vivo* Upper panel: Kinetics of tumor growth in a xenograft mouse model. TRIM28^WT^ and TRIM28^KD^ (sh#1) cells from the MDA-MB-231 **A.** and MCF-7 **B.** cell line were subcutaneously injected into athymic nude mice, and tumor size was measured weekly for 7-8 weeks. Error bars, SEM; **** p < 0.0001. Middle panel: *TRIM28* gene expression was downregulated in TRIM28^KD^ (sh#1) xenografts, as confirmed by RT-qPCR. Error bars, SD; * p < 0.05; **** p < 0.0001. Bottom panel: The image shows a representative group of MDA-MB-231 (A) and MCF-7 (B) tumors excised 7-8 weeks after injection.

Next, we decided to elucidate the role of TRIM28 protein in regulation of tumor growth *in vivo*. TRIM28-depleted and non-modified cells (WT) from selected breast cancer cell lines were injected subcutaneously into athymic nude mice (5 × 10^6^ cells/injection site, 12 animals per group). The growth kinetics of MDA-MB-231 and MCF-7 xenografts suggested that *TRIM28* depletion led to the inhibition of tumor growth in MDA-MB-231 cells (p = 1E-04), which have a high percentage of CD44^+^/CD24^−/low^ cells, but not in MCF-7, which have a very low number of CD44^+^/CD24^−/low^ cells (Figure 4, upper panel). We confirmed the downregulation of *TRIM28* gene expression in representative groups of MDA-MB-231 and MCF-7 xenografts excised 7-8 weeks after injection (Figure 4, middle and bottom panel). Additional T-47D luminal cancer cell line poor in CD44^+^/CD24^−/low^ population was also tested *in vivo* (Supplementary Figure S4) presenting no impact of *TRIM28* downregulation on xenograft growth. TRIM28-mediated regulation of tumor growth *in vivo* differs from *in vitro* observations.

### Depletion of *TRIM28* gene affects the cancer stem cell population and correlates with downregulation of mesenchymal markers

**Figure 5:**
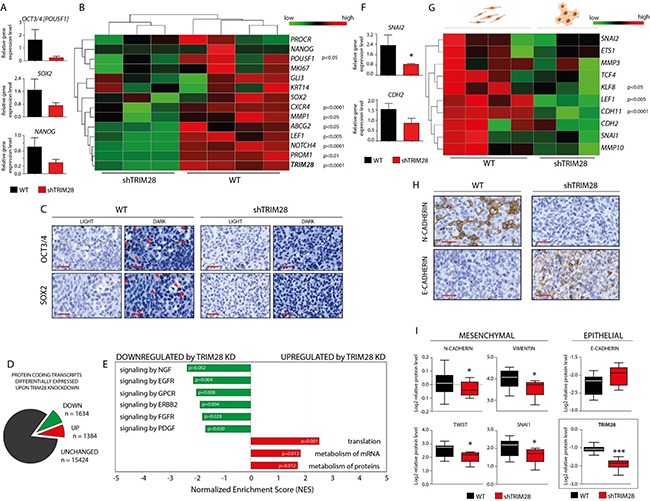
*TRIM28* knockdown leads to downregulation of pluripotency and mesenchymal markers and inhibition of stem cell-related pathways in MDA-MB-231 xenografts **A.** Box plots presenting relative expression of selected pluripotency markers in TRIM28^WT^ and TRIM28^KD^ (sh#1) xenografts evaluated using the RT-qPCR analysis. Error bars, SEM; p > 0.05. **B.** Heatmap of selected pluripotency markers expressed in xenografts based on RNA sequencing. *TRIM28* gene expression was efficiently downregulated in all of the TRIM28^KD^ (sh#1) samples, as shown at the bottom of the heatmap of pluripotency markers. Statistical significance (p-value) is presented in the figure. Green = downregulation; Red = upregulation. **C.** IHC staining confirmed downregulation of OCT3/4 and SOX2 pluripotency markers in TRIM28^KD^ (sh#1) MDA-MB-231 xenografts compared with TRIM28^WT^ tumors. Scale = 50 μm. **D.** Protein coding transcripts differentially expressed upon *TRIM28* knockdown. 1634 markers were significantly (FDR 5%) downregulated and 1384 markers were significantly upregulated in TRIM28^KD^ tumors when compared to TRIM28^WT^ xenografts. List of 3018 differentially expressed transcripts was further used for Gene Set Enrichment Analysis (GSEA). **E.** Summary of significantly changed (p < 0.05) gene sets after *TRIM28* knockdown identified using GSEA analysis. Signaling pathways involved in the regulation of stem cell phenotype were inhibited in TRIM28^KD^ (sh#1) xenografts. In contrast, gene sets that were upregulated in TRIM28^KD^ (sh#1) xenografts included several pathways involved in protein metabolism and translational regulation. **F.** Box plots presenting relative expression of selected mesenchymal markers in TRIM28^WT^ and TRIM28^KD^ (sh#1) xenografts evaluated using the RT-qPCR analysis. Error bars, SEM; * p < 0.05. **G.** Heatmap of selected mesenchymal markers expressed in xenografts based on RNA sequencing. Statistical significance (p-value) is presented in the figure. Green = downregulation; Red = upregulation. **H.** IHC staining confirmed the downregulation of N-CADHERIN (mesenchymal marker) and the upregulation of E-CADHERIN (epithelial marker) in TRIM28^KD^ (sh#1) MDA-MB-231 xenografts compared to TRIM28^WT^ tumors. Scale = 50 μm. **I.** TRIM28-dependant downregulation of selected mesenchymal markers and inhibition of “cadherin switch” was further confirmed using RPPA analysis. Error bars, SD; * p < 0.05.

Unlike MCF-7 cells, MDA-MB-231 cells are enriched in stem cell-like features [27, 30]. Therefore, we hypothesized that the inhibition of tumor growth in *TRIM28*-downregulated MDA-MB-231 cells is associated with the reduction of the CSC population. We tested the expression of the pluripotency markers *SOX2*, *OCT3/4* and *NANOG* in MDA-MB-231 xenografts using RT-qPCR. Indeed, the expression of these pluripotency markers was decreased in TRIM28-depleted xenografts (Figure 5A and 5B), suggesting that *TRIM28* downregulation led to a loss of stem cell properties. IHC staining confirmed the reduced levels of OCT3/4 and SOX2 transcription factors in TRIM28-depleted xenografts (Figure 5C).

To further explore the global changes in gene expression caused by *TRIM28* knockdown in MDA-MB-231 cell lines and xenografts compared to wild-type populations, global mRNA expression profiling was performed with next generation sequencing (Supplementary Table S2). As with the experimental phenotype, the differential expression profiles of the cell lines were significantly different from those of the xenografts (Supplementary Figure S5). As expected, the expression of selected pluripotency markers was lower in TRIM28^KD^ xenografts than in TRIM28^WT^ tumors (Figure 5B), [29, 31–36].

There were 3018 differentially expressed protein coding transcripts (5% FDR) upon *TRIM28* knockdown (Figure 5D). 1634 markers were significantly downregulated and 1384 markers were significantly upregulated in TRIM28^KD^ tumors when compared to TRIM28^WT^ xenografts. This also included several pluripotency genes known in the literature (*OCT3/4*, *CXCR4, MMP1*, *ABCG2*, *LEF1*, *NOTCH4*, *PROM1*), [29, 31–36]. Pathway analysis using GSEA identified 23 significantly changed gene sets after *TRIM28* depletion (Figure 5E and Supplementary Tables S3–S4). The gene sets downregulated in TRIM28^KD^ xenografts included signaling pathways involved in the maintenance of the stem cell phenotype (Supplementary Table S3), as previously described [37–44]. In contrast, the upregulated gene sets in TRIM28^KD^ xenografts included several pathways involved in mRNA metabolism and translational regulation (Supplementary Table S4).

Next, we checked whether this association of TRIM28 with pluripotency marker is observable in BRCA patients. Specifically, whether these markers have more coordinated expression in the more aggressive Basal subtypes compared to others. Therefore, we used ssGSEA with a curated set of pluripotency markers to score BRCA patient profiles [29, 31–36]. Indeed, the pluripotency genes differentially expressed upon *TRIM28* knockdown had increased concordant expression (ssGSEA score) in TCGA BRCA basal patients and better distinguished the highly aggressive basal subtype from the less aggressive luminal A subtype (Supplementary Figure S6). These results suggest that *TRIM28* expression correlates with the expression of selected cancer stem cell markers in breast cancer and indirectly implies that *TRIM28* has a role in BCSC regulation in patients.

We assessed whether inhibition of the epithelial-to-mesenchymal (EMT) transition, which results in the loss of pluripotency [4], might explain the decrease in tumor growth upon *TRIM28* knockdown. We found that the expression of EMT markers was decreased in TRIM28^KD^ xenografts, using RT-qPCR (Figure 5F), RNA-Seq (Figure 5G), immunohistochemistry (Figure 5H) and RPPA analysis (Figure 5I). RT-qPCR was performed for *CDH2* (encoding N-CADHERIN) and *SLUG*/*SNAI2*. A set of EMT markers was queried in the RNA-Seq profiles [45–50]. Moreover, IHC staining confirmed the downregulation of N-CADHERIN and upregulation of E-CADHERIN in TRIM28-depleted xenografts (Figure 5H). Furthermore, RPPA analysis of MDA-MB-231 xenografts confirmed downregulation of mesenchymal markers expression (VIMENTIN, N-CADHERIN, SNAI1 and TWIST) together with upregulation of epithelial marker E-CADHERIN in TRIM28^KD^ xenografts (Figure 5I). These results imply that TRIM28 protein might play a role in the EMT process and suggest that the inhibition of EMT might be the mechanism responsible for the loss of stem-cell properties.

### *TRIM28* knockdown reduces the number of cancer stem cells in MDA-MB-231 breast cancer xenografts

**Figure 6:**
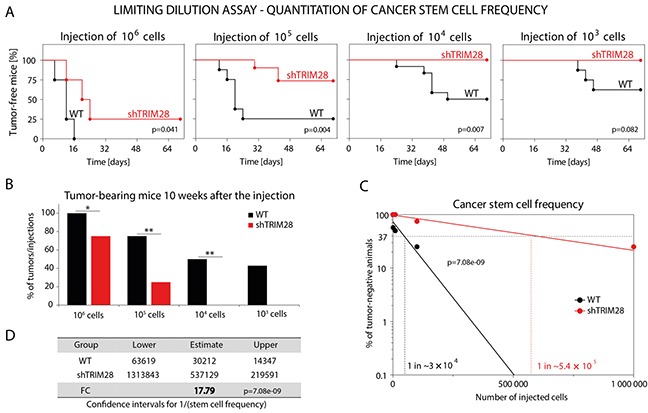
*TRIM28* gene depletion reduces the number of cancer stem cells in MDA-MB-231 breast cancer xenografts **A.** Limiting dilution assay was performed to estimate the hypothetical frequency of cancer stem cells in CD44^+^CD24^−/low^-enriched MDA-MB-231 breast cancer cell line upon *TRIM28* knockdown. TRIM28^WT^ and TRIM28^KD^ (sh#1) MDA-MB-231 cancer cells were injected subcutaneously in serial dilutions (10^6^, 10^5^, 10^4^ and 10^3^ of cells per injection) into athymic nude mice and the ability of cancer cells to induce tumor growth *in vivo* was examined for 10 weeks. *TRIM28* depletion (sh#1) reduced the ability of MDA-MB-231 breast cancer cells to induce tumor growth *in vivo*. **B.** Injection of TRIM28^KD^ MDA-MB-231 (sh#1) cells resulted in a reduced number of xenografts compared with TRIM28^WT^ cells 10 weeks after the engraftment. **C, D.** Hypothetical frequency of cancer stem cells in TRIM28^WT^ and TRIM28^KD^ (sh#1) MDA-MB-231 xenografts. *TRIM28* knockdown significantly diminished the number of cancer stem cells in MDA-MB-231 xenografts (FC = 17.79, p = 7.08e-09). The calculation of the estimated stem cell frequency for each condition was performed using ELDA software (ref. 51).

To further verify whether TRIM28-dependent inhibition of tumor growth and TRIM28-related downregulation of pluripotency markers expression are associated with a reduction in the CSC subpopulation, we performed a limiting dilution transplantation assay [51]. To analyze the ability of MDA-MB-231 TRIM28^WT^ and TRIM28^KD^ cells to induce tumor growth, several dilutions of cells were injected subcutaneously into athymic nude mice, and the appearance of tumors that were larger than 5 mm × 5 mm was monitored for 10 weeks. As expected, *TRIM28* depletion reduced the capacity of MDA-MB-231 cells to induce tumor growth, which implies that TRIM28 protein plays a role in the regulation of the CSC population (Figure 6A and 6B). Moreover, calculating the estimated stem cell frequency for each condition (WT vs. shTRIM28) revealed nearly 18 times more CSCs (FC = 17.79) in MDA-MB-231-TRIM28^WT^ cells than in TRIM28-depleted variant (p = 7.08E-09; Figure 6C and D). Altogether, these results confirmed the involvement of TRIM28 protein in the maintenance of the CSC population in breast cancer.

### TRIM28-dependent inhibition of triple-negative breast tumor growth could be mediated by “metabolic switch” from OXPHOS to GLYCOLYSIS in cancer cells

To elucidate the mechanism governing TRIM28-dependent inhibition of triple-negative breast tumor growth, the level of more than 300 protein markers was analyzed using RPPA platform at the MD Anderson core facility. We observed significant downregulation of 95 markers and upregulation of 24 markers in TRIM28^KD^ xenografts when compared with TRIM28^WT^ tumors (p < 0.05, FDR < 0.1; Supplementary Figure S7A). Several Reactome pathways were significantly downregulated (FDR < 0.1) in TRIM28^KD^ xenografts (Supplementary Figure S7B). Among others, *Cellular response to stress* (FDR = 3.19E-06), *Signal transduction* (FDR = 2.44E-04) and *Cell cycle* (FDR = 1.71E-03) events are highly downregulated upon *TRIM28* gene knockdown. Moreover, using a Cytoscape plugin BiNGO (The Biological Networks Gene Ontology tool) - an open-source Java tool to determine which Gene Ontology (GO) terms are significantly overrepresented in a set of markers, we further confirmed overrepresentation of terms associated with *Developmental processes*, *Cell cycle*, *Cellular response to stress* and *Signaling* in TRIM28^WT^ xenografts (Supplementary Figure S7C, Supplementary Figure S8). We also observed significant overrepresentation of *Metabolic processes* in TRIM28^WT^ xenografts, suggesting the involvement of metabolic events in the regulation of self-renewal of cancer stem cell population as previously reported by Viale *et al*. [10].

**Figure 7:**
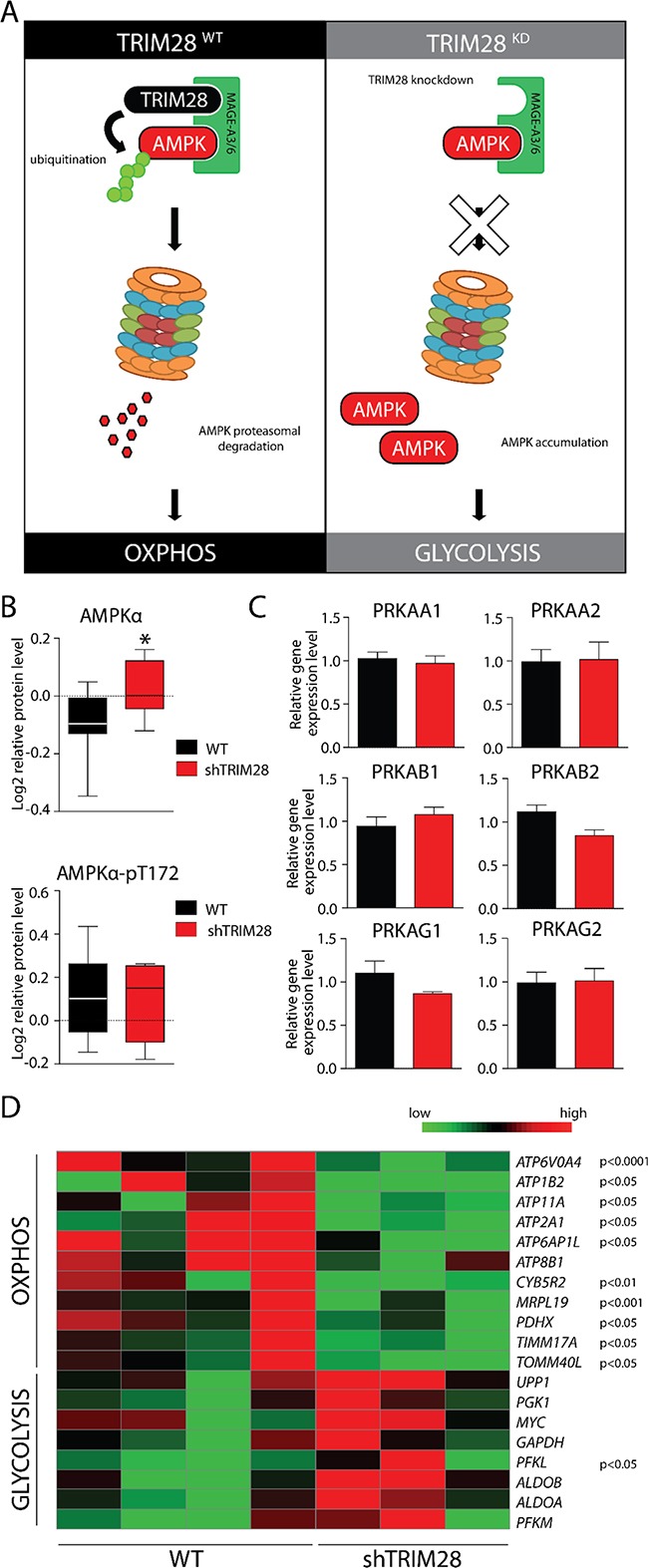
AMPK accumulation upon *TRIM28* knockdown mediates metabolic switch from OXPHOS to glycolysis in cancer cells **A.** MAGE-A3/6-TRIM28 cancer-specific ubiquitin ligase targets AMPKα kinase for proteasomal degradation in TRIM28^WT^ breast cancer cells. However, *TRIM28* knockdown (right panel) should shut down AMPKα ubiquitination and proteasomal degradation, resulting in AMPKα accumulation-mediated metabolic switch. **B.** Level of AMPKα and phospho-AMPKα (T172) in TRIM28^WT^ an TRIM28^KD^ xenografts was determined by RPPA analysis. Error bars, SD; * p < 0.05. **C.** AMPKα protein is composed of 3 independent subunits (α, β and γ) and each of AMPKα subunits is encoded by at least two gene isoforms. As presented on bar graphs, *TRIM28* downregulation does not affect the level of AMPK encoding genes as determined with RNA-Seq. Error bars, SD. **D.** Heatmap of selected metabolism-associated markers expressed in xenografts based on RNA sequencing. Statistical significance (p-value) is presented in the figure. Green = downregulation; Red = upregulation.

Moreover, TRIM28 was previously shown to form a cancer-specific ubiquitinase (together with MAGE-A3/6 proteins [52] and target AMPK, a master regulator of metabolic/energy homeostasis and mitochondrial biogenesis in cancer cells [53] for proteasomal degradation (Figure 7A). Indeed, we observed significant upregulation of total AMPK protein in RPPA profiles (Figure 7B). The level of phosphorylated AMPK (T172-phospho) was unchanged. *TRIM28* knockdown also didn't suppress the expression of AMPK-encoding genes (3 subunits: α, β and γ) which further confirms, that TRIM28 as an E3 ubiquitin ligase reduces the stability of AMPK protein (Figure 7C).

**Figure 8:**
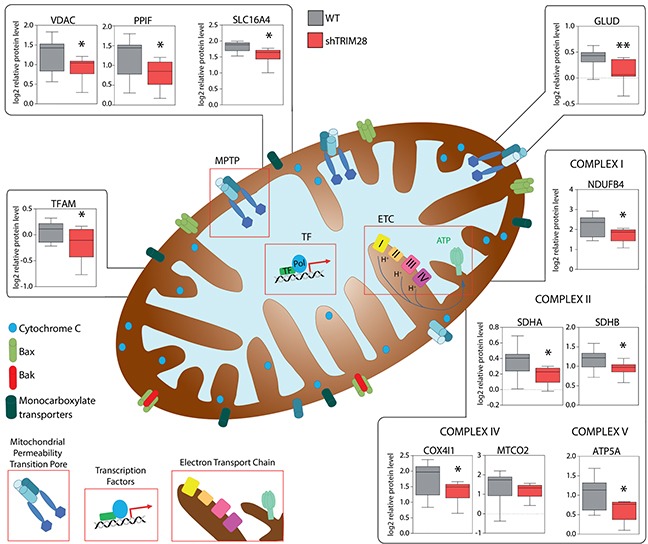
TRIM28-dependant inhibition of triple-negative breast tumor growth is mediated by metabolic changes/attenuation of oxidative phosphorylation in cancer cells RPPA analysis revealed significant downregulation of many proteins engaged in mitochondrial electron transport chain (ETC), formation of mitochondrial permeability transition pore (MPTP) and regulation of mitochondrial transcription suggesting that TRIM28-dependant inhibition of triple-negative breast tumor growth is mediated by metabolic changes in the tumor cells.

Next we checked expression levels of several markers for GLYCOLYSIS and OXPHOS curated based on Molecular Signatures Database (MSigDB) v5.1 [54]. Indeed, expression of GLYCOLYSIS markers was increased and expression of OXPHOS markers was decreased (Figure 7D) in TRIM28-downregulated xenografts. Lastly, we observed significant downregulation of proteins involved in Electron Transport Chain (ETC), formation of Mitochondrial Permeability Transition Pore (MPTP) as well as mitochondrial Transcription Factor TFAM. Therefore, it is attractive to conclude that downregulation of *TRIM28* may lead to accumulation of AMPK in cancer cells resulting in “metabolic switch” from oxidative phosphorylation (OXPHOS) to glycolysis (Figure 7A) and such metabolic dysregulation could be a reason for observed TRIM28-dependent inhibition of triple-negative breast tumor growth *in vivo* (Figure 8).

## DISCUSSION

The major finding of our study is novel role of TRIM28 protein in tumorigenesis of breast cancer through regulation of the self-renewal of CSC. TRIM28 is up-regulated in breast tumors, and high *TRIM28* expression is significantly associated with triple-negative breast tumors with stem cell-like features and poor survival. Recently, Trim28 protein has been reported to control the expression of pluripotency markers, such as *Oct3/4*, *Sox2* and *Nanog* in mouse embryonic stem cells (mESCs), [12, 13]. Depletion of *Trim28* resulted in significant downregulation of *Oct3/4*, *Sox2* and *Nanog* mRNA levels, which led to the differentiation of mESCs into the primitive ectoderm lineage. Together with other pluripotency markers (Cnot3, Zfx and c-Myc), Trim28 co-occupies many putative gene promoters and forms a unique module in the self-renewal transcription network. Moreover, TRIM28 protein is involved in the transcriptional activation of EMT program, which is linked to the acquisition of stem cell properties in breast cancer [18]. Furthermore, *TRIM28* was the most differentially expressed gene in axitinib-sensitive cancer cell lines [55]. Axitinib is known to target cancer stem-like cells, which further supports our hypothesis that TRIM28 plays a role in the maintenance of the CSC population [56].

Therefore, we proposed that *TRIM28* depletion in breast cancer cell lines may inhibit self-renewal of CSCs and sensitize the cells to standard therapies. It seems that aggressive breast tumors often enriched in CSCs [33, 50, 57], exhibit moderate to strong nuclear positivity for TRIM28 and TRIM28-S824-phospho protein more frequently than luminal A tumors, considered to be more differentiated and nonaggressive (Figure 1D). Although these results are not statistically significant, probably due to small number of tested samples, they are consistent with previous reports [22, 23], (see also Supplementary Table S1). TRIM28 protein expression positively correlates with tumor aggressiveness, which may indicate TRIM28 association with a stem-like phenotype. To date only one paper has demonstrated tumor growth inhibition *in vivo* upon *TRIM28* knockdown, however, the exact mechanism of TRIM28-dependent tumor growth inhibition remains to be elucidated [21].

Previously, Addison *et al*. [24] demonstrated that *TRIM28* gene depletion led to inhibition of MDA-MB-231 cell proliferation *in vitro*. The authors counted cells for 8 days and demonstrated that *TRIM28* knockdown inhibited cell proliferation only after prolonged period of cell culture. The authors observed no difference after 24 hours of cell growth. In our studies we observed that *TRIM28* downregulation has no impact on cell proliferation and viability *in vitro* in all tested breast cancer cell lines (Figure 2F and 2G, Figure 3F-3H and Supplementary Figure S2C and S2D) 24 hours after seeding [24]. We observed that *TRIM28* knockdown did not affect CD44^+^CD24^−/low^ population *in vitro* in all tested cell lines (Figure 2H and Supplementary Figure S2E). At the same time, RT-qPCR analysis indicated no difference in the expression of pluripotency markers *OCT3/4*, *SOX2* and *NANOG* in *TRIM28*-downregulated cells versus TRIM28^WT^ cells (Figure 2I) suggesting no impact of TRIM28 KD in regulation of cancer cells *in vitro* at early time points. Therefore, based on our results and recently published data we suggest that *TRIM28* knockdown has little effect on cell proliferation *in vitro*. It is also in-line with the fact that TRIM28 has an “epigenetic” role as opposed to signaling role, which manifests in earlier phenotypes.

TRIM28 protein participates in the DDR pathway [17, 58]. Upon DNA damage, ATM kinase phosphorylates TRIM28 throughout the nucleus, leading to local chromatin decondensation and creating a chromatin configuration that is essential for a fully efficient repair process [17, 59]. Moreover, in unstressed cells TRIM28 represses the expression of pro-apoptotic genes: *TP53AIP1*, *BAX*, *BBC3* (PUMA) and *PMAIP1* (NOXA), [60, 61]. Furthermore, TRIM28 inhibits p53 activation and together with MDM2, promotes p53 proteosomal degradation [15]. In short, TRIM28 provides a survival advantage to cells by contributing to the transcriptional repression of the DDR genes and inactivation of p53 [15, 59, 61–63].

Therefore, we investigated whether *TRIM28* knockdown would sensitize breast cancer cells to DNA-damaging agents: doxorubicin or γ-irradiation *in vitro.* A previous report regarding another drug, actinomycin D, demonstrated significant sensitization of breast cancer TRIM28-depeleted cells to chemotherapeutic agents compared with non-modified cells [15]. Unexpectedly, our results indicated that *TRIM28* downregulation did not affect cell proliferation and viability after doxorubicin treatment *in vitro* (Figure 3A-3D). Such diversity in results may be due to the mechanism of drug action. Actinomycin D targets cell proliferation by inhibiting transcription and indirectly stabilizing p53 through the sequestration of MDM2 to the nucleolus (inactivation of MDM2 E3-ligase activity), [16]. Therefore, as shown by Okamoto *et al*. [15], sensitization of breast cancer cells to actinomycin D after *TRIM28* knockdown might result from the simultaneous inactivation of both MDM2 and TRIM28. This assumption is confirmed by the fact that Okamoto *et al*. [15] did not observe similar results for another agent, camptothecin. Moreover, it was previously demonstrated that *TRIM28* downregulation in the U2-OS human osteosarcoma cell line sensitizes these cells to neocarzinostatin, an ionizing radiation mimetic [17]. Similarly, TRIM28-depleted HT1080 cells were more radiosensitive than the control cells, which might suggest TRIM28-dependent sensitization to γ-irradiation. However, the genetic backgrounds of U2-OS cells and HT1080 cells differ significantly from those selected for our study [17]. In our studies, we did not observe increased apoptosis after irradiation in TRIM28-depleted cells *in vitro* (Figure 3E). Hence, we suggest that the divergence in cell-specific response, the mechanism of drug action and the sensitivity of the assay used to analyze cell proliferation or viability together might explain the observed differences between our findings and the results of other researchers.

Cancer stem cells possess the ability to migrate and initiate new tumor lesions in distal location [29, 64]. Our migration studies *in vitro* (Figure 3I) show that very aggressive MDA-MB-231 triple-negative breast cancer cells scarcely migrate *in vitro* in contrast to control lung cancer cell line H1299. Additionally, using the mammosphere formation assay of selected breast cancer cell lines demonstrated that the fibroblastic-like cell line MDA-MB-231 formed small loosely adherent structures, which rather resemble cell clumps [65, 66] than the mammospheres that could be cultured past passage 2 or 3. Therefore, we discontinued further *in vitro* analyses and focused our efforts using the *in vivo* experiments.

It should be noted that specific properties of cancer stem cells are maintained not only by the endogenous signaling pathways (i.e. Notch, Shh, Wnt/β-catenin) and other complex biological processes (i.e. the EMT) but also by the external environment called the niche, while the lack of signals from the niche can lead to loss of self-renewal capacity [5–7]. Therefore, it seemed more appropriate to analyze the impact of *TRIM28* gene depletion on breast cancer stem cell population in mouse model *in vivo*. In contrast to *in vitro* data, we observed a significant inhibition of tumor growth *in vivo* in MDA-MB-231 cells (95-98% CD44^+^/CD24^−/low^; relatively high level of *OCT3/4*) in contrast to MCF-7 or T-47D cells (1-10% CD44^+^/CD24^−/low^; relatively low level of *OCT3/4*) upon TRIM28 knockdown (Figure 4 and Supplementary Figure S4). Based on transcriptomic and proteomic analyses, we further investigated TRIM28-dependent inhibition of triple-negative breast tumor growth *in vivo*. We have focused on high-throughput RNA-Seq and RPPA assays due to limited size of TRIM28-depleted MDA-MB-231 xenografts. Performed analyses allowed us to measure broad panel of markers associated with breast cancer stem cells.

Our results demonstrate that *TRIM28* knockdown led to the downregulation of pluripotency markers (Figure 5A-5C), suggesting that tumor growth inhibition is due to reduction in the CSC pluripotency. Furthermore, *TRIM28* depletion led to downregulation of mesenchymal markers (Figure 5F-5I), which provides evidence for inhibition of the EMT, one of the mechanisms for maintaining the CSC population [4, 44, 46]. Moreover, the RNA-Seq results and IHC staining validated our finding that *TRIM28* downregulation led to the loss of tumor cells' pluripotency. The gene sets that were reduced in TRIM28^KD^ MDA-MB-231 xenografts (Figure 5E and Supplementary Table S3) represent signaling pathways involved in breast cancer cell proliferation and controlling the complex mechanism of stem cell maintenance [37–44]. Previously, Descamps *et al*. [41] demonstrated that NGF is a strong stimulator of breast cancer cell proliferation. Moreover, cell signaling through G-protein coupled receptors (GPCR signaling pathway) has been reported to play a major role in stem cell biology [39]. Similarly, several lines of evidence have indicated that HER2 (ERBB2) is an important positive regulator of the CSC population in HER2+ breast cancers and other tumors. As reported by Ithimakin *et al*. [42] in luminal breast cancers that do not display HER2 amplification, HER2 is selectively expressed in and drives the CSC population. Furthermore, EGFR signaling stimulates the self-renewal and expansion of stem-like cells, and inhibition of EGFR pathway resulted in significant downregulation of SOX2 pluripotency marker and decreased self-renewal capability *in vitro* [37]. Moreover, the involvement of FGFR signaling in stem cell self-renewal is recognized [44], although the mechanisms by which FGF maintains stemness are poorly defined. Together, these signaling pathways are the critical components of the CSC regulatory network; therefore, significant downregulation in their activity after *TRIM28* knockdown in MDA-MB-231 xenografts suggests that TRIM28 protein has a crucial role in CSC homeostasis *in vivo*.

Furthermore, the functional assay for CSC identification *in vivo*, the limiting dilution transplantation assay, revealed a TRIM28-dependent reduction in the CSC population in xenografts from the MDA-MB-231 cell line (FC = 17.79; p = 7.08E-09). Compared with TRIM28^WT^ cells, the ability of TRIM28-depleted cells to induce tumor growth was highly reduced (Figure 5A and 5B). Similar results were obtained by Leis *et al*. [67] after *SOX2* knockdown in breast cancer cell lines. Downregulation of pluripotency marker *SOX2* expression led to inhibited tumor growth and a reduced capacity of cancer cells to induce tumor growth when injected subcutaneously in several serial dilutions. Our results are in line with the effect presented by Leis *et al*. [67], indicating TRIM28 as a positive modulator of CSC population.

Furthermore, based on RPPA analysis in MDA-MB-231 xenografts, we observed significant downregulation of proteins engaged in the regulation of cell cycle, response to cellular stress, developmental processes and mitochondrial functions in TRIM28^KD^ (sh#1) xenografts (Supplementary Figure S4 and Supplementary Figure S5).

Regulation of cell cycle was previously linked with the maintenance of stemness. Strikingly, S and G2 phase-associated pathways were demonstrated to trigger selective preference toward pluripotency maintenance when the progression of stem cell through the cell cycle was perturbed. In their work, Gonzales *et al*. [8] revealed that ATM/ATR-CHEK2-p53 and Cyclin B1 pathways are employed in the S and G2 phases of the cell cycle, respectively, to inhibit stem cell differentiation. Furthermore, Singh *et al*. [68] demonstrated that stem cells in late G1 phase are prone to initiate differentiation from this period of the cell cycle. They showed that bivalently marked developmental genes, possessing both active and repressive histone marks, are only transcribed during the late G1 phase in human ESCs due to cell cycle dependent recruitment of the histone methyltransferase complex subunit MLL2, which further confirm involvement of cell cycle regulating machinery in the maintenance of stem cell pluripotency. Moreover, expression of higher levels of quiescence and dormancy-associated genes, including *CDKN1B* and *CHEK1*, was observed in highly metastatic breast cancer cells considered to be tumor-initiating cells (TICs) with stem-like properties [64].

We have detected significant depletion of proteins involved in cell cycle regulation and response to stress signals through ATM kinase (Supplementary Figure S5). Our results are in agreement with previously reported data indicating cell cycle dependent maintenance of stem cell pluripotency.

Among others, a set of genes involved in oxidative phosphorylation (OXPHOS) is coordinately decreased in *TRIM28* depleted tumors (Figure 7 and Figure 8). Viale *et al*. [10] presented recently that cancer stem cells have increased mitochondrial activity when compared to bulk cells. Transcriptomic and metabolic analyses of cells identified as pancreatic cancer stem cells revealed prominent expression of genes governing mitochondrial function, autophagy and lysosome activity, as well as a strong reliance on mitochondrial respiration and a decreased dependence on glycolysis for cellular energetics. Moreover, Pasto *et al*. [69] presented the metabolic profile of CSCs characterized by preferential fueling of glucose into oxidative phosphorylation and the pentose phosphate pathway. Consistently, De Luca *et al*. [70] demonstrated that XCT790 – a selective inverse agonist ligand of the estrogen-related receptor alpha (ERRα), markedly reduced the oxidative phosphorylation in breast cancer cells and suppressed the activity of several signaling pathways that are normally required for the survival of CSCs, leading to inhibition of breast cancer stem cell propagation as determined with mammosphere formation assay. Moreover, the AMPK kinase - a master regulator of metabolic/energy homeostasis and mitochondrial biogenesis in cancer cells, mediates the “metabolic switch” from oxidative phosphorylation (OXPHOS) to glycolysis [53] and Pineda and Potts [52] recently demonstrated that TRIM28 together with MAGE-A3/6 proteins form a cancer-specific ubiquitinase that target AMPK for proteasomal degradation. In our data we observed significant upregulation of AMPK protein level in TRIM28-depleted xenografts, suggesting loss of function of cancer-specific TRIM28-MAGE-A3/6 ubiquitinase (Figure 7).

The importance of mitochondrium-dependent modulation of stem cell self-renewal was further supported by Katajisto *et al*. [69] who demonstrated that during stem cell asymmetric division the daughter cells that receive mainly “young” mitochondria maintained stem cell traits, while old organelles are segregated to differentiating progeny.

We have shown that downregulation of *TRIM28* expression results profound changes in breast cancer xenografts including: loss of pluripotency and mesenchymal markers, inhibition of stem cell-associated signaling pathways, reduced ability to induce tumor growth and reduced number of cancer stem cells. We conclude that impaired mitochondrial functions and “metabolic switch” from OXPHOS to glycolysis results in loss of self-renewal of breast cancer stem cell and lead to tumor growth inhibition of upon *TRIM28* knockdown. However, the exact direct mechanism of TRIM28-mediated regulation of CSC metabolism in breast cancer remains to be elucidated. We believe that our findings may shed new light on the epigenetic hemostasis of breast cancer stem cells and pave the way to novel and more effective therapies that target TRIM28 protein in breast tumors.

## MATERIALS AND METHODS

Investigation has been conducted in accordance with the ethical standards and according to the Declaration of Helsinki and according to national and international guidelines and has been approved by the authors' institutional review board.

### Cell culture

The original cell lines were obtained from American Type Culture Collection (ATCC, Manassas, VA, USA). MDA-MB-231 (ER-, PR-, HER2-, TP53^mut^), MDA-MB-468 (ER-, PR-, HER2-, TP53^mut^), H1299 (lung cancer) and HEK-293T cells were grown in Dulbecco's Modified Eagle's Medium (DMEM), 5 g/L of glucose, with 10% fetal bovine serum (FBS), 50 units/ml penicillin, and 50 μg/ml streptomycin (all from Invitrogen, Carlsbad, CA, USA). MCF-7 (ER+, PR+, HER2-, TP53^WT^), T-47D (ER+, PR+, HER2-, TP53^mut^), Hs-578T (ER-, PR-, HER2-, TP53^mut^) and BT-549 (ER-, PR-, HER2-, TP53^mut^) cells were cultured in the same medium with the addition of 0.01 mg/ml human recombinant insulin in a humidified atmosphere at 37°C, 21% O_2_ and 5% CO_2_. The stable TRIM28-knockdown cell lines and control cells transfected with an empty vector were grown as described above with the addition of 1 μg/ml puromycin (Sigma-Aldrich, St. Louis, MO, USA). Additional culture conditions were used for selected cell lines: hypoxia – 1% O2, serum-restricted conditions: 0.5%, 1% and 2% of FBS as well as glucose-restricted conditions: 0.5, 0.05 and 0 g/L.

### Lentiviral vector production and stable TRIM28^KD^ cell line preparation

To produce lentiviral vectors (LV-shTRIM28 and control vector LV-puro-ctrl), HEK-293T cells were co-transfected with psPAX2, pMD2.G and lentiviral plasmid pWPTS-shTRIM28 (#1 or #2) or pWPTS-puro-ctrl. The culture supernatant was collected 48 hours post transfection and passed through 0.45-μm filters, and aliquots were stored at −80°C. All breast cancer cell lines were infected with lentiviruses, and 1 μg/ml puromycin was added 72 hours after infection. The cells were selected using puromycin (Sigma) for 8-10 days and were subsequently tested for *TRIM28* expression.

### Western blot analysis

Whole cell lysates were prepared by lysing the cells with radioimmune precipitation assay (RIPA) buffer (Sigma) plus Complete Protease Inhibitor Mixture (Roche Applied Science, Indianapolis, IN, USA) and were subjected to SDS-PAGE followed by immunoblotting with antibodies for TRIM28 (ab10483 or ab10484, Abcam, Cambridge, MA, USA) and β-actin (ab75168, Abcam). The blots were visualized using an enhanced chemiluminescence detection kit (ECL-Plus, Amersham Biosciences, Piscataway, NJ, USA) and a G:BOX F3 Gel Documentation System (Syngene). The results of the western blot analyses shown in this report are representative of four independent experiments.

### Total RNA extraction, reverse transcription and RT-qPCR

Total RNA was extracted using TRI Reagent RNA Isolation Reagent (Sigma) according to the manufacturer's protocol. Reverse transcription was performed using the iScript cDNA Synthesis Kit (Bio-Rad Laboratories, Inc., Hercules, CA, USA) with 1 μg of total RNA for reaction. Gene-specific primers and probes were used for real-time qPCR. PCR amplification and fluorescence detection were performed using a Light Cycler 480 Real-Time PCR detection system (Roche), and the threshold cycles were determined using Light Cycler 480 Software. Fold inductions were determined using the ΔΔCt method against the GAPDH gene.

### FACS analysis

The cells were washed twice with PBS, harvested with 2 mM EDTA (Invitrogen) and resuspended in ice-cold PBS (0.7 × 10^6^ cells/100 μl). Combinations of fluorochrome-conjugated monoclonal antibodies against human CD44 (anti-CD44-APC; cat. #559942) and CD24 (anti-CD24-PE; cat. #555428) were obtained from BD Biosciences (San Diego, CA, USA). The primary antibodies or the respective isotype controls (BD Biosciences) were added to the cell suspension, as recommended by the manufacturer, and were incubated at 4°C in the dark for 30 min. The labeled cells were analyzed using a FACSCanto analyzer (BD Biosciences).

### Cell proliferation assay and chemotherapy

The cells were seeded into 96-well plates to obtain a confluency of 50% on the day of the experiment. The cells were treated with vehicle or serial dilutions of doxorubicin (doxorubicin hydrochloride, Sigma; range, 0-1 μM) for 24, 48 or 72 hours. A 20-μl aliquot of full medium containing 1 μCi of ^3^H-thymidine (specific activity 70-90 Ci/mmol (2590-3330 GBq/mmol, Perkin Elmer) was added to each well 16-18 hours before the termination of culture by freezing. Incorporated ^3^H-thymidine was assessed using a Micro Beta TriLux scintillation counter (Perkin Elmer, Waltham, MA, USA). Simultaneously, cell viability *in vitro* was measured using a standard ATPlite™ Luminescence Assay (Perkin Elmer).

### Radiotherapy

The cells were irradiated using a 6-MV accelerating potential on a Varian Clinac 2300 linear accelerator with a dose range of 0 Gy to 8 Gy. The proliferation potential was evaluated using a ^3^H-thymidine-incorporation assay.

### Migration assay

Cell migration experiments were carried out using the xCELLigence® RTCA DP instrument (Roche Diagnostics GmbH, Mannheim, Germany) which was placed in a humidified incubator at 37°C, 21% O_2_ and 5% CO_2_. Modified 16-well plates (CIM-16, Roche Diagnostics GmbH, Mannheim, Germany) with each well consisting of an upper and a lower chamber separated by a microporous membrane containing randomly distributed 8 μm-pores were used according to manufacturer's protocol. Prior to each experiment, cells were deprived of FBS during 24 hours. 10% FBS containing culture medium was used as a chemoattractant. Cell migration was analyzed for 48 hours after seeding.

### Immunostaining

Immunohistochemical studies were performed on breast cancer surgical specimens using the avidin-biotin-peroxidase method (DakoCytomation, Carpinteria, CA, USA) on formalin-fixed, paraffin-embedded tissues (FFPE). All of the sections were counterstained using hematoxylin. The product names and the dilutions of primary antibodies against the specific markers used in the study are available upon request.

### Tumor growth in vivo

The experiments were approved by the Local Ethical Committee for Experiments on Animals in Poznan; the animals were maintained according to the standards established by the Ministry of Agriculture and Rural Development in Poland (2006). Female 6- to 8-week-old athymic nude mice (12 animals per group) were injected with 5 × 10^6^ non-modified or shTRIM28-expressing cells. For MCF-7 and T-47D xenografts, controlled 17β-estradiol pellets (60 days release 0.72 mg; Innovative Research of America, Sarasota, FL, USA) were transplanted behind the neck one week before the cell injection. The cells were washed and harvested in PBS and were subcutaneously injected in a 0.1-mL volume into the flanks of mice. The tumors were measured with caliper, and the volumes (V) were calculated as follows:

$$V=\frac{1}{2}*L*W^{2},$$

where L is tumor length and W is tumor width.

After 7-8 weeks, the mice were sacrificed, and the tumors were excised, cut into sections and prepared for further analyses.

### Limiting dilution transplantation assay

Female 6- to 8-week-old athymic nude mice were injected with serial dilutions of non-modified or shTRIM28-expressing MDA-MB-231 breast cancer cells. The cells were washed and harvested in PBS and were subcutaneously injected in a 0.1-mL volume into the flanks of mice. The mice were monitored every 4 days for tumor growth (at least 5 × 5 mm). The calculation of the estimated stem cell frequency for each condition was performed using ELDA software as described previously [51].

### RNA-Seq analysis

The RNA from cell lines and xenografts was isolated using TRI Reagent solution according to the manufacturer's protocol. The RNA integrity number (RIN) was assessed using BioAnalyzer2000 (Agilent) and the samples that met the criteria for RNA-Seq analysis (RIN ≥ 9) were analyzed at the Institute for Applied Cancer Science, MD Anderson Cancer Center (Houston, TX, USA), as previously described [71–73]. Briefly, cDNA was synthesized from mRNA samples, converted into double-stranded DNA and then subjected to library preparation using the Illumina TruSeq^TM^ RNA sample preparation kit (low-throughput protocol) according to the manufacturer's protocol. Between 50 million and 90 million purity filtered reads were obtained per sample using an Illumina Hi-Seq sequencer. The raw reads were aligned to human reference genome assembly version GRCh37 using Bowtie2. The samples were quality controlled using FASTQC and RNA-Se-QC. The exon profiling efficiency in each case was more than 80%, suggesting very high-quality data. The raw expression counts were obtained using the Bioconductor package easyRNA-Seq and ENSEMBL annotations. The counts were normalized and the differential expression was identified using the Bioconductor DESeq package [74, 75]. The DESeq package utilizes negative binomial distribution to call differential expression. We did not observe length-related bias in differentially expressed genes. Genes that showed a ≥1.3-fold change in expression and at least 1% FDR were termed differentially expressed genes. The gene set enrichment analysis was conducted using the GSEA pre-ranked method.

### Reverse phase protein array (RPPA)

The cells were washed twice with PBS, then lysed in RPPA lysis buffer: 1% Triton X-100, 50 mM HEPES, pH 7.4, 150 mM NaCl, 1.5 mM MgCl_2_, 1 mM EGTA, 100 mM NaF, 10 mM Na_4_O_7_P_2_, 1 mM Na_3_VO_4_, 10% glycerol containing freshly added 1% protease inhibitor cocktail (Sigma-Aldrich) and 1% phosphatase inhibitor cocktail (Sigma-Aldrich). After 30-minute incubation on ice, protein lysates were centrifuged for 30 min. at 12000 rpm and supernatants were collected. Protein concentration was determined by BCA assay and adjusted to 1.5 μg/μl. Each sample was mixed with 4 · SDS Sample Buffer (40% Glycerol, 8% SDS, 0.25M Tris-HCL, pH 6.8; with 10% 2-mercaptoethanol) and incubated for 5 minutes at 95°C. RPPA analyses were performed at the RPPA core facility at MD Anderson Cancer Center (Houston, USA) using standard operating procedures and the panel of more than 300 highly validated antibodies.

### Statistical analysis of TCGA and other data

TCGA breast invasive carcinoma RNA-Seq data and associated clinical information for more than 1000 patients were downloaded from the Firehose genome data analysis center (available at http://gdac.broadinstitute.org), standard data release 2014_07_15. PAM50 subtype information was available for 950 of these patients. All of the patients were scored for gene sets from MSigDB version 4.0 (available at www.broad.mit.edu/gsea/msigdb/) using GSEA software. Literature-curated lists of pluripotency, epithelial and mesenchymal markers were used to score the patients using single sample gene set analysis (ssGSEA) projection algorithm to identify concordant expression of these gene sets in patients with BRCA. The survival analysis was conducted using R-package “survival,” and other clinical traits were associated with TRIM28 expression using the chi-squared test, Wilcox test and Kruskal-Wallis test, as applicable.

## SUPPLEMENTARY FIGURES AND TABLES





## References

[1] Visvader JE, Lindeman GJ (2012). Cancer stem cells: Current status and evolving complexities. Cell Stem Cell.

[2] Czerwinska P, Kaminska B (2015). Regulation of breast cancer stem cell features. Contemp Oncol.

[3] Muñoz P, Iliou MS, Esteller M (2012). Epigenetic alterations involved in cancer stem cell reprogramming. Mol Oncol.

[4] Mani SA, Guo W, Liao MJ, Eaton EN, Ayyanan A, Zhou AY, Brooks M, Reinhard F, Zhang CC, Shipitsin M, Campbell LL, Polyak K, Brisken C (2008). The epithelial-mesenchymal transition generates cells with properties of stem cells. Cell.

[5] Li L, Neaves WB (2006). Normal stem cells and cancer stem cells: The niche matters. Cancer Res.

[6] Lu H, Clauser KR, Tam WL, Fröse J, Ye X, Eaton EN, Reinhardt F, Donnenberg VS, Bhargava R, Carr SA, Weinberg RA (2014). A breast cancer stem cell niche supported by juxtacrine signalling from monocytes and macrophages. Nat Cell Biol.

[7] Brooks MD, Wicha MS (2015). Tumor Twitter: Cellular Communication in the Breast Cancer Stem Cell Niche. Cancer Discov.

[8] Gonzales KA, Liang H, Lim YS, Chan YS, Yeo JC, Tan CP, Gao B, Le B, Tan ZY, Low KY, Liou YC, Bard F, Ng HH (2015). Deterministic Restriction on Pluripotent State Dissolution by Cell-Cycle Pathways. Cell.

[9] Ito K, Suda T (2014). Metabolic requirements for the maintenance of self-renewing stem cells. Nat Rev Mol Cell Biol.

[10] Viale A, Pettazzoni P, Lyssiotis CA, Ying H, Sánchez N, Marchesini M, Carugo A, Green T, Seth S, Giuliani V, Kost-Alimova M, Muller F, Colla S (2014). Oncogene ablation-resistant pancreatic cancer cells depend on mitochondrial function. Nature.

[11] Friedman JR, Fredericks WJ, Jensen DE, Speicher DW, Huang XP, Neilson EG, Rauscher FJ (1996). KAP-1, a novel corepressor for the highly conserved KRAB repression domain. Genes Dev.

[12] Hu G, Kim J, Xu Q, Leng Y, Orkin SH, Elledge SJ (2009). A genome-wide RNAi screen identifies a new transcriptional module required for self-renewal. Genes Dev.

[13] Seki Y, Kurisaki A, Watanabe-Susaki K, Nakajima Y, Nakanishi M, Arai Y, Shiota K, Sugino H, Asashima M (2010). TIF1beta regulates the pluripotency of embryonic stem cells in a phosphorylation-dependent manner. Proc Natl Acad Sci U S A.

[14] Santoni de Sio FR, Massacand J, Barde I, Offner S, Corsinotti A, Kapopoulou A, Bojkowska K, Dagklis A, Fernandez M, Ghia P, Thomas JH, Pinschewer D, Harris N (2012). KAP1 regulates gene networks controlling mouse B-lymphoid cell differentiation and function. Blood.

[15] Okamoto K, Kitabayashi I, Taya Y (2006). KAP1 dictates p53 response induced by chemotherapeutic agents via Mdm2 interaction. Biochem Biophys Res Commun.

[16] Poyurovsky MV, Jacq X, Ma C, Karni-Schmidt O, Parker PJ, Chalfie M, Manley JL, Prives C (2003). Nucleotide binding by the MDM2 RING domain facilitates Arf-independent MDM2 nucleolar localization. Mol Cell.

[17] Ziv Y, Bielopolski D, Galanty Y, Lukas C, Taya Y, Schultz DC, Lukas J, Bekker-Jensen S, Bartek J, Shiloh Y (2006). Chromatin relaxation in response to DNA double-strand breaks is modulated by a novel ATM- and KAP-1 dependent pathway. Nat Cell Biol.

[18] Venkov CD, Link AJ, Jennings JL, Plieth D, Inoue T, Nagai K, Xu C, Dimitrova YN, Rauscher FJ, Neilson EG (2007). A proximal activator of transcription in epithelial-mesenchymal transition. J Clin Invest.

[19] Yang Y, Fiskus W, Yong B, Atadja P, Takahashi Y, Pandita TK, Wang HG, Bhalla KN (2013). Acetylated hsp70 and KAP1-mediated Vps34 SUMOylation is required for autophagosome creation in autophagy. Proc Natl Acad Sci U S A.

[20] Cufí S, Vazquez-Martin A, Oliveras-Ferraros C, Martin-Castillo B, Vellon L, Menendez JA (2011). Autophagy positively regulates the CD44(+) CD24(-/low) breast cancer stem-like phenotype. Cell Cycle.

[21] Gong C, Bauvy C, Tonelli G, Yue W, Deloménie C, Nicolas V, Zhu Y, Domergue V, Marin-Esteban V, Tharinger H, Delbos L, Gary-Gouy H, Morel AP (2012). Beclin 1 and autophagy are required for the tumorigenicity of breast cancer stem-like/progenitor cells. Oncogene.

[22] Yokoe T, Toiyama Y, Okugawa Y, Tanaka K, Ohi M, Inoue Y, Mohri Y, Miki C, Kusunoki M (2010). KAP1 is associated with peritoneal carcinomatosis in gastric cancer. Ann Surg Oncol.

[23] Yu C, Zhan L, Jiang J, Pan Y, Zhang H, Li X, Pen F, Wang M, Qin R, Sun C (2014). KAP-1 is overexpressed and correlates with increased metastatic ability and tumorigenicity in pancreatic cancer. Med Oncol.

[24] Addison JB, Koontz C, Fugett JH, Creighton CJ, Chen D, Farrugia MK, Padon RR, Voronkova MA, McLaughlin SL, Livengood RH, Lin CC, Ruppert JM, Pugacheva EN (2014). KAP1 Promotes Proliferation and Metastatic Progression of Breast Cancer Cells. Cancer Res.

[25] Stickeler E, Pils D, Klar M, Orlowsk-Volk M, Zur Hausen A, Jäger M, Watermann D, Gitsch G, Zeillinger R, Tempfer CB (2011). Basal-like molecular subtype and HER4 up-regulation and response to neoadjuvant chemotherapy in breast cancer. Oncol Rep.

[26] Györffy B, Lanczky A, Eklund AC, Denkert C, Budczies J, Li Q, Szallasi Z (2010). An online survival analysis tool to rapidly assess the effect of 22,277 genes on breast cancer prognosis using microarray data of 1,809 patients. Breast Cancer Res Treat.

[27] Neve RM, Chin K, Fridlyand J, Yeh J, Baehner FL, Fevr T, Clark L, Bayani N, Coppe JP, Tong F, Speed T, Spellman PT, DeVries S (2009). A collection of breast cancer cell lines for the study of functionally. Cancer Cell.

[28] Prat A, Karginova O, Parker JS, Fan C, He X, Bixby L, Harrell JC, Roman E, Adamo B, Troester M, Perou CM (2013). Characterization of cell lines derived from breast cancers and normal mammary tissues for the study of the intrinsic molecular subtypes. Breast Cancer Research and Treatment.

[29] Sheridan C, Kishimoto H, Fuchs RK, Mehrotra S, Bhat-Nakshatri P, Turner CH, Goulet R, Badve S, Nakshatri H (2006). CD44+/CD24- breast cancer cells exhibit enhanced invasive properties: an early step necessary for metastasis. Breast Cancer Res.

[30] Ricardo S, Vieira AF, Gerhard R, Leitão D, Pinto R, Cameselle-Teijeiro JF, Milanezi F, Schmitt F, Paredes J (2011). Breast cancer stem cell markers CD44, CD24 and ALDH1: expression distribution within intrinsic molecular subtype. J Clin Pathol.

[31] Band V, Zhao X, Malhotra G, Band H (2011). Shared signaling pathways in normal and breast cancer stem cells. J Carcinog.

[32] Al-Dhfyan A (2013). Embryonic signature in breast cancers; Pluripotency roots of cancer stem cells. Saudi Pharm J.

[33] Milla LA, González-Ramírez CN, Palma V (2012). Sonic hedgehog in cancer stem cells: A novel link with autophagy. Biol Res.

[34] Britton KM, Eyre R, Harvey IJ, Stemke-Hale K, Browell D, Lennard TW, Meeson AP (2012). Breast Cancer, Side Population cells and ABCG2 expression. Cancer Lett.

[35] Hwang-Verslues WW, Kuo WH, Chang PH, Pan CC, Wang HH, Tsai ST, Jeng YM, Shew JY, Kung JT, Chen CH, Lee EY, Chang KJ, Lee WH (2009). Multiple lineages of human breast cancer stem/progenitor cells identified by profiling with stem cell markers. PLoS One.

[36] Liu H, Kato Y, Erzinger SA, Kiriakova GM, Qian Y, Palmieri D, Steeg PS, Price JE (2012). The role of MMP-1 in breast cancer growth and metastasis to the brain in a xenograft model. BMC Cancer.

[37] Singh S, Trevino J, Bora-Singhal N, Coppola D, Haura E, Altiok S, Chellappan SP (2012). EGFR/Src/Akt signaling modulates Sox2 expression and self-renewal of stem-like side-population cells in non-small cell lung cancer. Mol Cancer.

[38] Zhang W, Tan W, Wu X, Poustovoitov M, Strasner A, Li W, Borcherding N, Ghassemian M, Karin M (2013). A NIK-IKKα Module Expands ErbB2-Induced Tumor-Initiating Cells by Stimulating Nuclear Export of p27/Kip1. Cancer Cell.

[39] Callihan P, Mumaw J, Machacek DW, Stice SL, Hooks SB (2011). Regulation of stem cell pluripotency and differentiation by G protein coupled receptors. Pharmacol Ther.

[40] Fillmore CM, Gupta PB, Rudnick JA, Caballero S, Keller PJ, Lander ES, Kuperwasser C (2010). Estrogen expands breast cancer stem-like cells through paracrine FGF/Tbx3 signaling. Proc Natl Acad Sci U S A.

[41] Descamps S, Pawlowski V, Révillion F, Hornez L, Hebbar M, Boilly B, Hondermarck H, Peyrat JP (2001). Expression of nerve growth factor receptors and their prognostic value in human breast cancer. Cancer Res.

[42] Ithimakin S, Day KC, Malik F, Zen Q, Dawsey SJ, Bersano-Begey TF, Quraishi AA, Ignatoski KW, Daignault S, Davis A, Hall CL, Palanisamy N, Heath AN (2013). HER2 drives luminal breast cancer stem cells in the absence of HER2 amplification: Implications for efficacy of adjuvant trastuzumab. Cancer Res.

[43] Del Vecchio CA, Jensen KC, Nitta RT, Shain AH, Giacomini CP, Wong AJ (2012). Epidermal Growth Factor Receptor Variant III Contributes to Cancer Stem Cell Phenotypes in Invasive Breast Carcinoma. Cancer Res.

[44] Pond AC, Bin X, Batts T, Roarty K, Hilsenbeck S, Rosen JM (2013). Fibroblast Growth Factor Receptor Signaling Is Essential for Normal Mammary Gland Development and Stem Cell Function. Stem Cells.

[45] Sarrió D, Rodriguez-Pinilla SM, Hardisson D, Cano A, Moreno-Bueno G, Palacios J (2008). Epithelial-mesenchymal transition in breast cancer relates to the basal-like phenotype. Cancer Res.

[46] Blick T, Hugo H, Widodo E, Waltham M, Pinto C, Mani SA, Weinberg RA, Neve RM, Lenburg ME, Thompson EW (2010). Epithelial Mesenchymal Transition Traits in Human Breast Cancer Cell Lines Parallel the CD44hi/CD24lo/− Stem Cell Phenotype in Human Breast Cancer. J Mammary Gland Biol Neoplasia.

[47] Sarrio D, Franklin CK, Mackay A, Reis-Filho JS, Isacke CM (2012). Epithelial and Mesenchymal Subpopulations Within Normal Basal Breast Cell Lines Exhibit Distinct Stem Cell/Progenitor Properties. Stem Cells.

[48] Fan F, Samuel S, Evans KW, Lu J, Xia L, Zhou Y, Sceusi E, Tozzi F, Ye XC, Mani SA, Ellis LM (2012). Overexpression of Snail induces epithelial–mesenchymal transition and a cancer stem cell–like phenotype in human colorectal cancer cells. Cancer Med.

[49] May CD, Sphyris N, Evans KW, Werden SJ, Guo W, Mani SA (2011). Epithelial-mesenchymal transition and cancer stem cells: a dangerously dynamic duo in breast cancer progression. Breast Cancer Res.

[50] Findlay VJ, Wang C, Watson DK, Camp ER (2014). Epithelial to mesenchymal transition and the cancer stem cell phenotype: Insights from cancer biology with therapeutic implications for colorectal cancer. Cancer Gene Ther.

[51] Hu Y, Smyth GK (2009). ELDA: Extreme limiting dilution analysis for comparing depleted and enriched populations in stem cell and other assays. J Immunol Methods.

[52] Pineda CT, Potts PR (2015). Oncogenic MAGEA-TRIM28 ubiquitin ligase downregulates autophagy by ubiquitinating and degrading AMPK in cancer. Autophagy.

[53] Chaube B, Bhat MK (2016). AMPK, a key regulator of metabolic/energy homeostasis and mitochondrial biogenesis in cancer cells. Cell Death Dis.

[54] Subramanian A, Tamayo P, Mootha VK, Mukherjee S, Ebert BL, Gillette MA, Paulovich A, Pomeroy SL, Golub TR, Lander ES, Mesirov JP (2005). Gene set enrichment analysis: A knowledge-based approach for interpreting genome-wide expression profiles. Proc Natl Acad Sci.

[55] Garnett MJ, Edelman EJ, Heidorn SJ, Greenman CD, Dastur A, Lau KW, Greninger P, Thompson IR, Luo X, Soares J, Liu Q, Iorio F, Surdez D (2012). Systematic identification of genomic markers of drug sensitivity in cancer cells. Nature.

[56] Wang F, Mi YJ, Chen XG, Wu XP, Liu Z, Chen SP, Liang YJ, Cheng C, To KK, Fu LW (2012). Axitinib Targeted Cancer Stemlike Cells to Enhance Efficacy of Chemotherapeutic Drugs via Inhibiting the Drug Transport Function of ABCG2. Mol Med.

[57] Ben-Porath I, Thomson MW, Carey VJ, Ge R, Bell GW, Regev A, Weinberg RA (2008). An embryonic stem cell-like gene expression signature in poorly differentiated aggressive human tumors. Nat Genet.

[58] Wang C, Rauscher FJ, Cress WD, Chen J (2007). Regulation of E2F1 function by the nuclear corepressor KAP1. J Biol Chem.

[59] Cann KL, Dellaire G (2011). Heterochromatin and the DNA damage response: the need to relax. Biochem Cell Biol.

[60] Tian C, Xing G, Xie P, Lu K, Nie J, Wang J, Li L, Gao M, Zhang L, He F (2009). KRAB-type zinc-finger protein Apak specifically regulates p53-dependent apoptosis. Nat Cell Biol.

[61] Li X, Lee YK, Jeng JC, Yen Y, Schultz DC, Shih HM, Ann DK (2007). Role for KAP1 serine 824 phosphorylation and sumoylation/desumoylation switch in regulating KAP1-mediated transcriptional repression. J Biol Chem.

[62] Li X, Lin HH, Chen H, Xu X, Shih H-M, Ann DK (2010). SUMOylation of the transcriptional co-repressor KAP1 is regulated by the serine and threonine phosphatase PP1. Sci Signal.

[63] Lee YK, Thomas SN, Yang AJ, Ann DK (2007). Doxorubicin down-regulates Krüppel-associated box domain-associated protein 1 sumoylation that relieves its transcription repression on p21 WAF1/CIP1 in Breast cancer MCF-7 cells. J Biol Chem.

[64] Lawson DA, Bhakta NR, Kessenbrock K, Prummel KD, Yu Y, Takai K, Zhou A, Eyob H, Balakrishnan S, Wang CY, Yaswen P, Goga A, Werb Z (2015). Single-cell analysis reveals a stem-cell program in human metastatic breast cancer cells. Nature.

[65] Grimshaw MJ, Cooper L, Papazisis K, Coleman JA, Bohnenkamp HR, Chiapero-Stanke L, Taylor-Papadimitriou J, Burchell JM (2008). Mammosphere culture of metastatic breast cancer cells enriches for tumorigenic breast cancer cells. Breast Cancer Res.

[66] Manuel Iglesias J, Beloqui I, Garcia-Garcia F, Leis O, Vazquez-Martin A, Eguiara A, Cufi S, Pavon A, Menendez JA, Dopazo J, Martin AG (2013). Mammosphere Formation in Breast Carcinoma Cell Lines Depends upon Expression of E-cadherin. PLoS One.

[67] Leis O, Eguiara A, Lopez-Arribillaga E, Alberdi MJ, Hernandez-Garcia S, Elorriaga K, Pandiella A, Rezola R, Martin AG (2012). Sox2 expression in breast tumours and activation in breast cancer stem cells. Oncogene.

[68] Singh AM, Sun Y, Li L, Zhang W, Wu T, Zhao S, Qin Z, Dalton S (2015). Cell-Cycle Control of Bivalent Epigenetic Domains Regulates the Exit from Pluripotency. Stem Cell Reports.

[69] Pastò A, Bellio C, Pilotto G, Ciminale V, Silic-Benussi M, Guzzo G, Rasola A, Frasson C, Nardo G, Zulato E, Nicoletto MO, Manicone M, Indraccolo S (2014). Cancer stem cells from epithelial ovarian cancer patients privilege oxidative phosphorylation, and resist glucose deprivation. Oncotarget.

[70] De Luca A, Fiorillo M, Peiris-Pagès M, Ozsvari B, Smith DL, Sanchez-Alvarez R, Martinez-Outschoorn UE, Cappello AR, Pezzi V, Lisanti MP, Sotgia F (2015). Mitochondrial biogenesis is required for the anchorage- independent survival and propagation of stem-like cancer cells. Oncotarget.

[71] Mortazavi A, Williams BA, McCue K, Schaeffer L, Wold B (2008). Mapping and quantifying mammalian transcriptomes by RNA-Seq. Nat Meth.

[72] Samur MK, Yan Z, Wang X, Cao Q, Munshi NC, Li C, Shah PK (2013). canEvolve: A Web Portal for Integrative Oncogenomics. PLoS One.

[73] Anders S, Huber W (2010). Differential expression analysis for sequence count data. Genome Biol.

[74] Anders S, McCarthy DJ, Chen Y, Okoniewski M, Smyth GK, Huber W, Robinson MD (2013). Count-based differential expression analysis of RNA sequencing data using R and Bioconductor. Nat Protoc.

[75] Barbie DA, Tamayo P, Boehm JS, Kim SY, Moody SE, Dunn IF, Schinzel AC, Sandy P, Meylan E, Scholl C, Fröhling S, Chan EM, Sos ML (2009). Systematic RNA interference reveals that oncogenic KRAS-driven cancers require TBK1. Nature.

