# Supplementary material for: TRIM28 multi-domain protein regulates cancer stem cell population in breast tumor development
Source: Oncotarget. 2016 Nov 10;8(1):863–82. doi: 10.18632/oncotarget.13273 (PMC5352203; doi:10.18632/oncotarget.13273)
Supplement: Supplementary file 1 [file oncotarget-08-863-s001.pdf]

TRIM28 multi-domain protein regulates cancer stem cell population in breast tumor development

SUPPLEMENTARY FIGURES AND TABLES

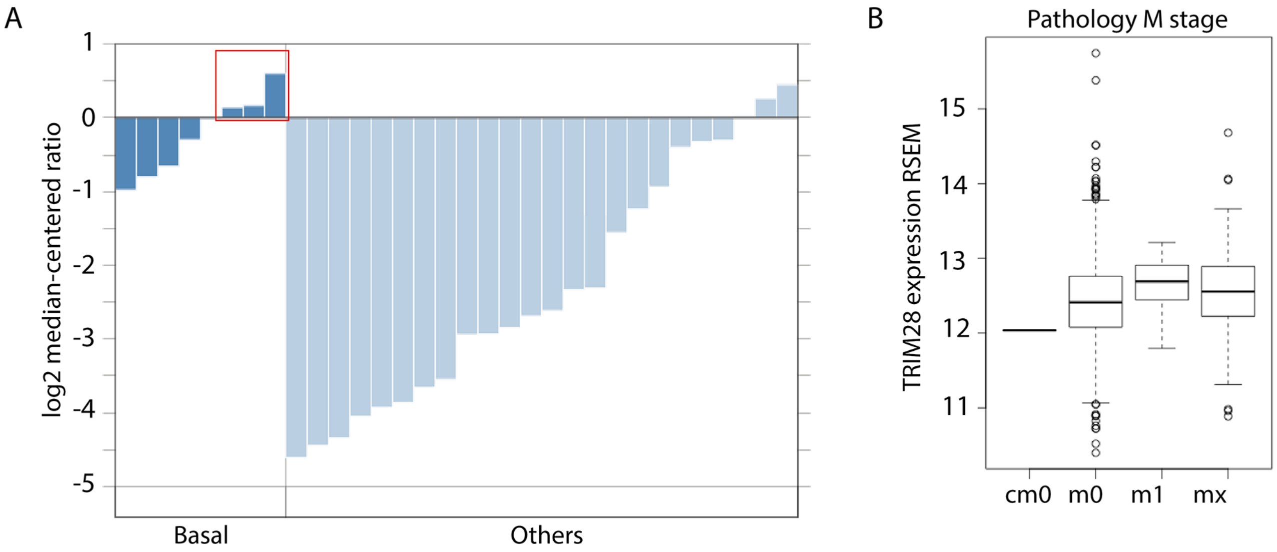

Supplementary Figure S1: Association of *TRIM28* expression with aggressive breast cancer. A. Elevated expression of *TRIM28* gene in basal (containing triple-negative BRCA) compared to other subtypes of breast cancer. **B.** *TRIM28* gene expression is distinct between different pathology M stages with the highest expression level in pathology M1 stage.

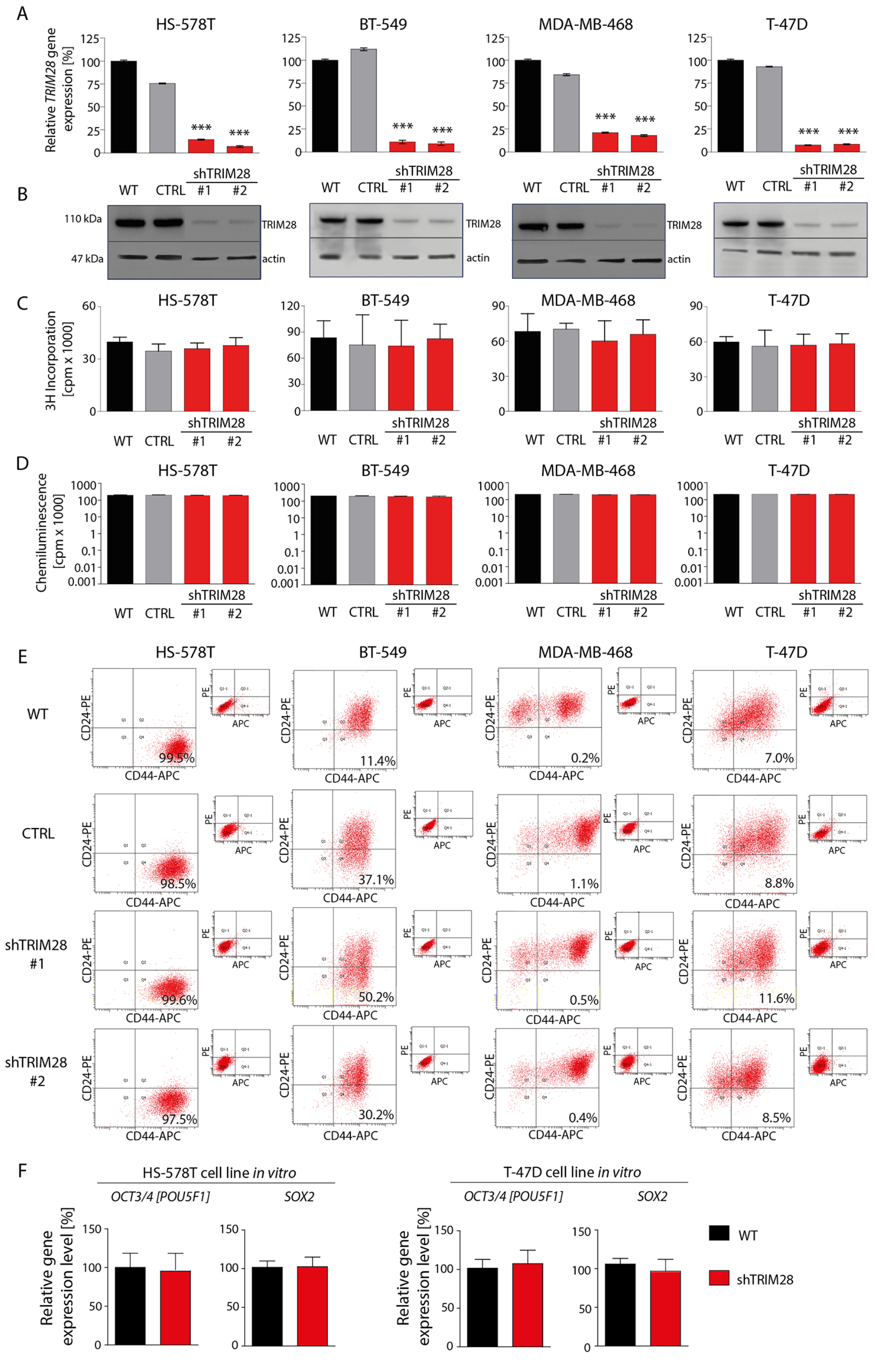

Supplementary Figure S2: shRNA-mediated knockdown of *TRIM28* gene in a panel of 4 breast cancer cell lines. A, B. After the transduction with TRIM28 shRNA-mediating vectors, the cells from all four cell lines exhibited a decreased expression of *TRIM28* confirmed by RT-qPCR (A) and Western blot analysis (B). CTRL-empty vector. Error bars, SD; n = 3. **C, D.** *TRIM28* downregulation does not affect cell proliferation (C) and viability (D) *in vitro* as determined using an ^{3}H-thymidine-incorporation assay and ATPlite™ Luminescence Assay, respectively. Error bars, SD; n = 4; p > 0.05. **E.** The comparison of CD44^{+}/CD24$^{-/low}$ cells frequency in breast cancer cell lines upon reduction of TRIM28 level *in vitro*. *TRIM28* knockdown does not affect the percentage of breast cancer stem cell population *in vitro*. **F.** *TRIM28* downregulation does not affect the expression of pluripotency markers *OCT3/4*, *SOX2* and *NANOG in vitro* in HS-578T (left panel) and T-47D (right panel) breast cancer cell lines as determined using RT-qPCR. Error bars, SD; n = 4; p > 0.05.

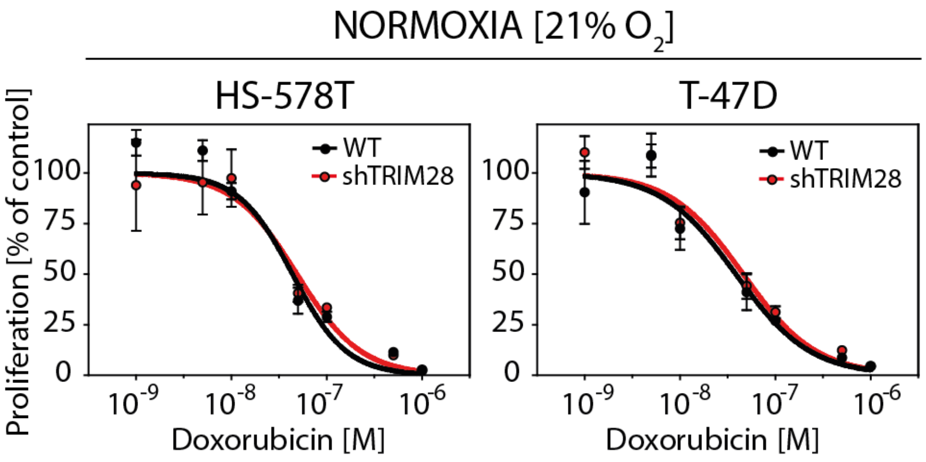

Supplementary Figure S3: *TRIM28* knockdown does not affect the chemoresistance of HS-578T and T-47D breast cancer cell lines *in vitro*. The dose response curves show the relative proliferation *in vitro* (^{3}H-thymidine incorporation assay) of TRIM28^{WT} and TRIM28^{KD} (sh#1) cells after doxorubicin treatment in normoxia. Error bars, SD; n = 4; p > 0.05.

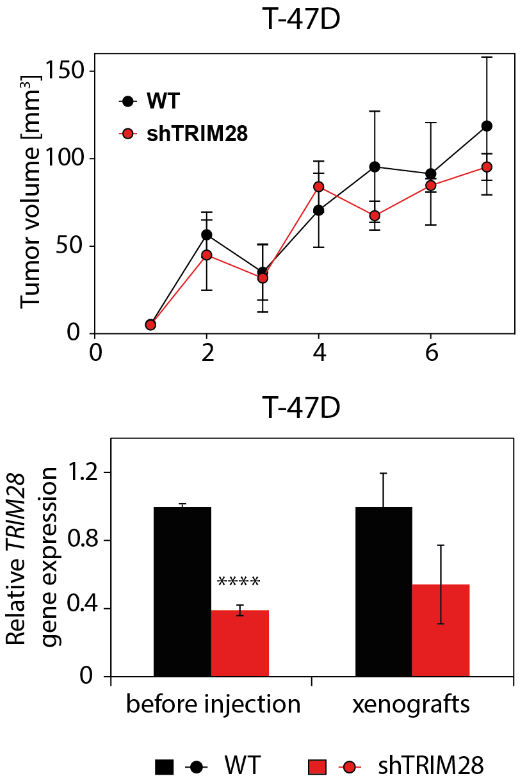

Supplementary Figure S4: Kinetics of T-47D tumor growth in a xenograft mouse model. Upper panel: TRIM28^{WT} and TRIM28^{KD} (sh#1) cells were subcutaneously injected into athymic nude mice, and tumor size was measured weekly for 7 weeks. Error bars, SEM. Bottom panel: *TRIM28* gene expression was downregulated in TRIM28^{KD} (sh#1) xenografts, as confirmed by RT-qPCR. Error bars, SD; **** p < 0.0001.

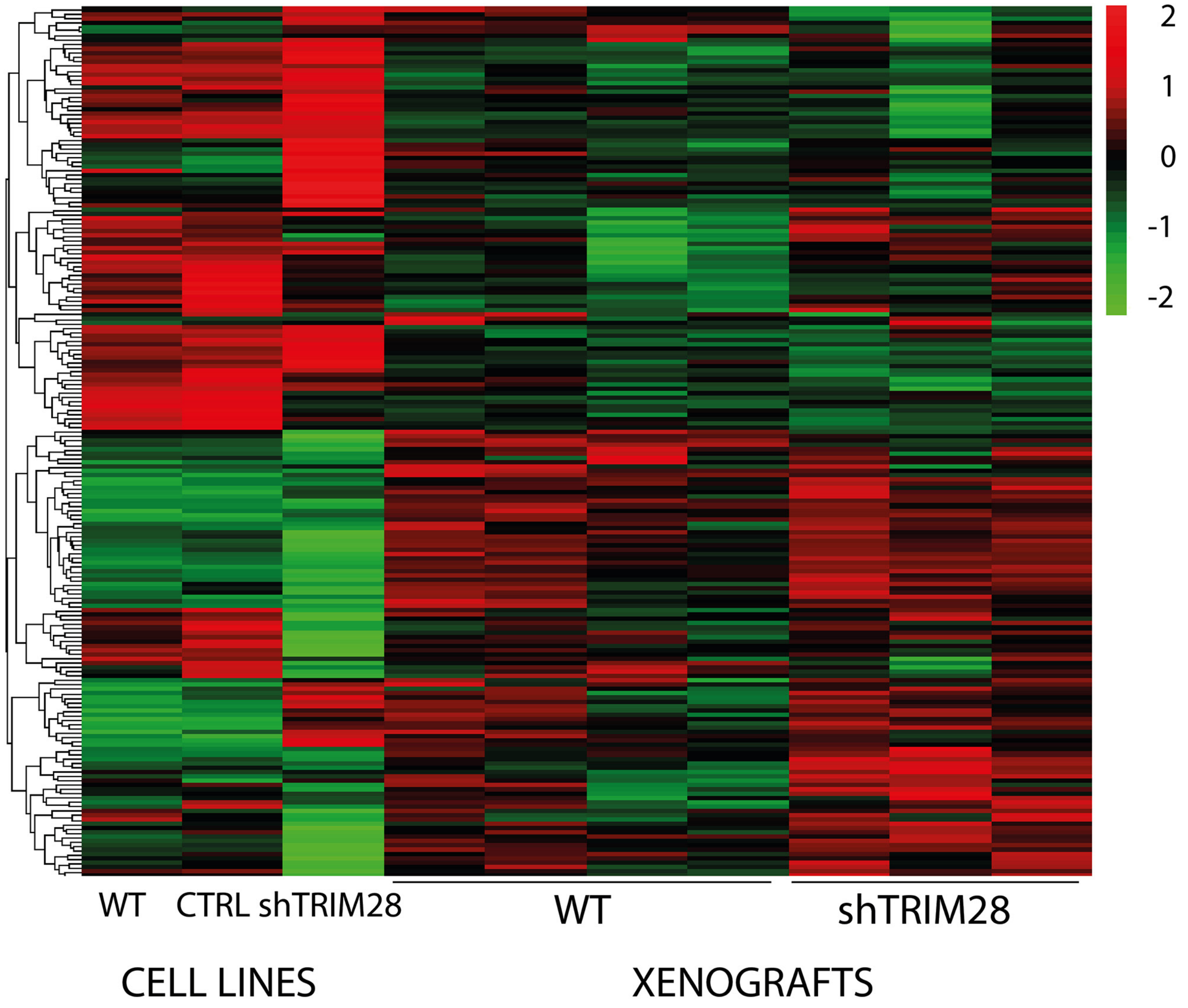

Supplementary Figure S5: Heatmap of 200 top differentially expressed genes between MDA-MB-231 xenografts and MDA-MB-231 cell lines. WT − non-modified cells/xenografts from non-modified cells; CTRL − MDA-MB-231 cells transduced with empty vector; shTRIM28 − MDA-MB-231 cells transduced with shTRIM28 encoding vector/xenografts from MDA-MB-231 cells with *TRIM28* knockdown.

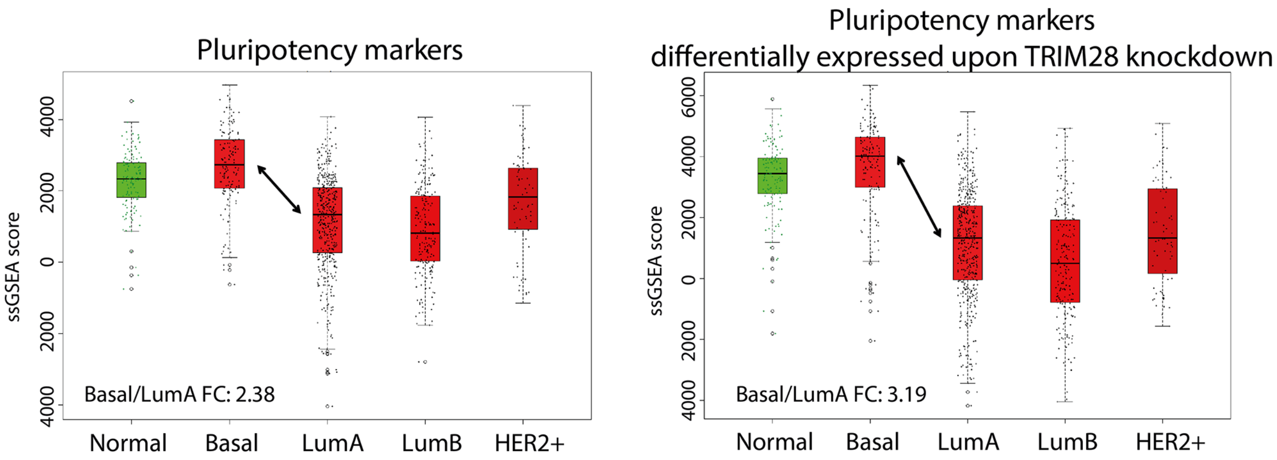

Supplementary Figure S6: Pluripotency markers differentially expressed upon *TRIM28* knockdown have increased concordant expression in TCGA BRCA basal subtype. A. Expression of selected pluripotency markers differs between intrinsic breast cancer subtypes. **B.** Six pluripotency markers differentially expressed upon *TRIM28* knockdown in xenografts (*OCT3/4*, *CXCR4*, *MMP1*, *ABCG2*, *LEF1*, *NOTCH4*, *PROM1*) have increased concordant expression in basal BRCA subtype and better distinguish highly aggressive basal to less aggressive luminal A subtype.

A

Proteins differentially expressed in TRIM28^{KD} xenografts vs wild-type tumors

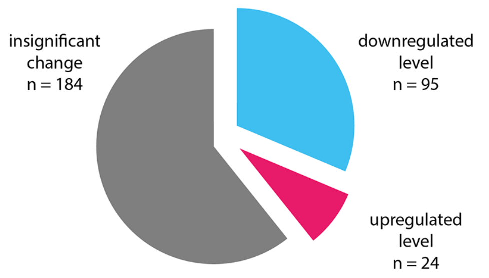

B

Reactome events downregulated in TRIM28^{KD} xenografts

Reactome event/pathway	Number of IDs in pathway	FDR
Cellular responses to stress	24/446	3,19E-06
Signal Transduction	60/2678	2,44E-04
Hemostasis	20/564	8,69E-04
Cell Cycle	20/598	1,71E-03
DNA Repair	10/185	1,99E-03
Disease	29/1193	8,55E-03
Gene Expression	33/1433	9,26E-03
Programmed Cell Death	8/179	1,67E-02
DNA Replication	6/118	2,93E-02
Cell-Cell communication	6/143	4,68E-02
Developmental Biology	17/801	9,20E-02
Immune System	32/1749	9,31E-02

C

Gene Ontology (GO) terms significantly overrepresented in a set of proteins upregulated in TRIM28^{WT} xenografts

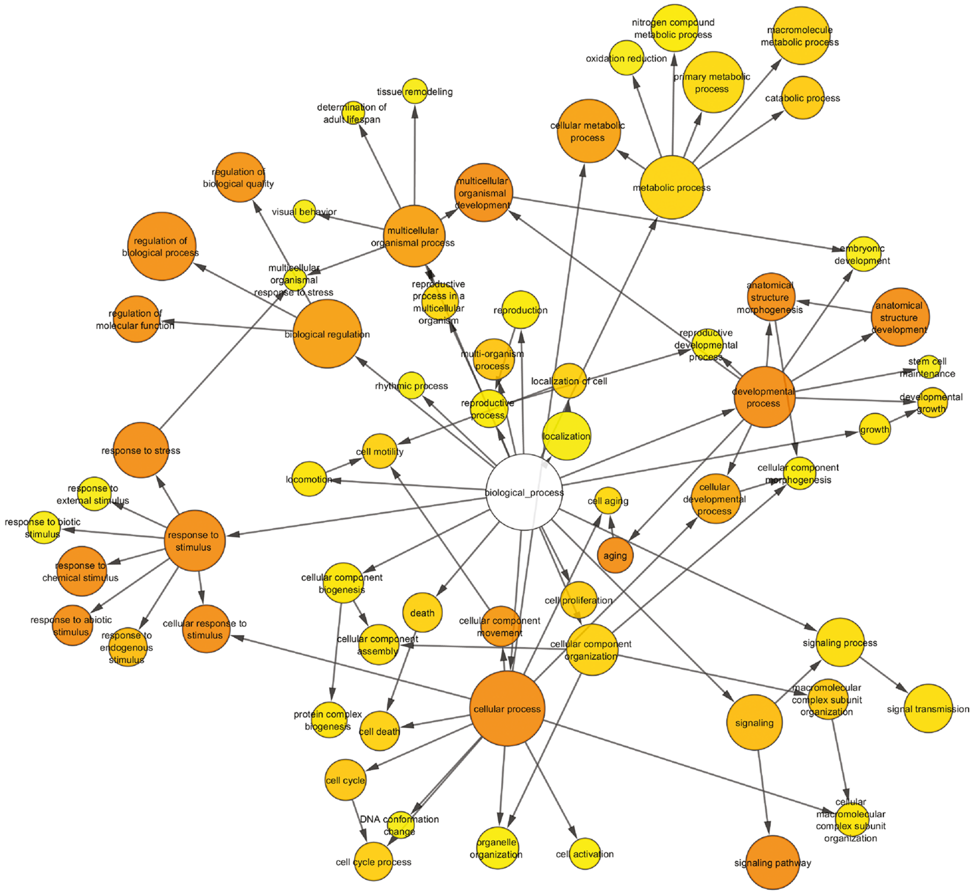

Supplementary Figure S7: RPPA analysis revealed significant downregulation of events/mechanisms associated with cell cycle, cellular response to stress, signal transduction and metabolism upon *TRIM28* gene knockdown. A. Statistical analysis of RPPA results indicated a significant downregulation of 95 markers (p-val < 0.05, FDR < 0.1) and upregulation of 24 markers (p-val < 0.05, FDR < 0.1) in TRIM28^{KD} (sh#1) xenografts when compared with TRIM28^{WT} tumors. **B.** The biological events which are significantly downregulated in TRIM28^{KD} (sh#1) xenografts (FDR < 0.1) as determined using a free, open-source, curated and peer reviewed pathway database Reactome (www.reactome.org). **C.** Analysis of RPPA data with a Cytoscape plugin BiNGO (The Biological Networks Gene Ontology tool) confirmed overrepresentation of terms associated with Developmental processes, Cell cycle, Cellular response to stress and Signaling in TRIM28^{WT} xenografts. Interestingly, overrepresentation of Metabolic processes in TRIM28^{WT} tumors was observed.

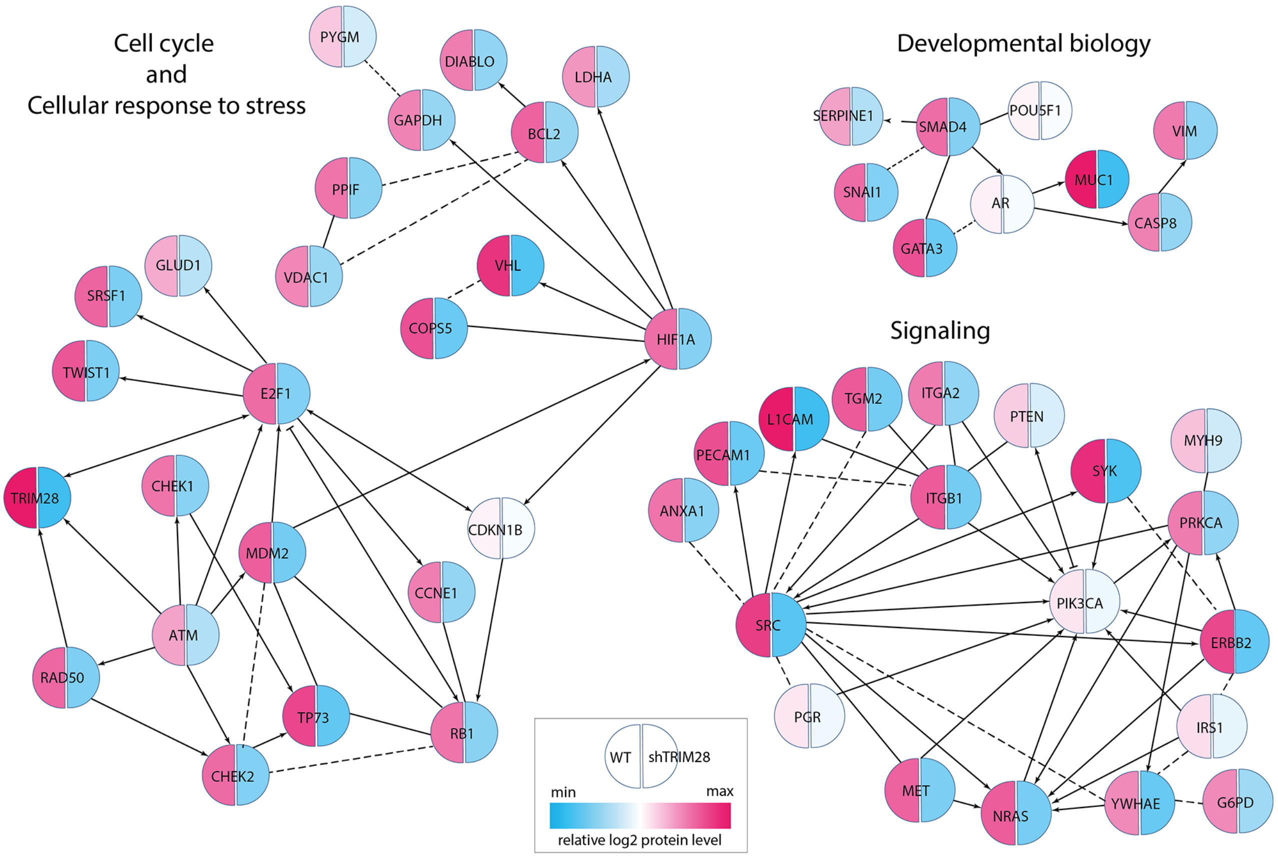

Supplementary Figure S8: Functional Interaction Network of significantly differentially expressed markers between TRIM28^{WT} and TRIM28^{KD} xenografts based on RPPA data. Visualization was prepared in Cytoscape ReactomeFIViz application. Blue = relative downregulation of protein level; Pink = relative upregulation of protein level.

Supplementary Table S1: Summary of *TRIM28* differential expression analysis from Oncomine database.

See Supplementary File 1

Supplementary Table S2: Summary of the RNA-Seq expression profiles of MDA-MB-231 cell lines and xenografts generated with control and TRIM28^{KD} conditions.

No.	ID	CELL LINE	CONDITION	TOTAL PURITY FILTERED READS
1	IACS-TRIM-18	MDA-MB-231	Breast Cancer Cell Line WT *In Vitro*	70217876
2	IACS-TRIM-19	MDA-MB-231	Breast Cancer Cell Line with Vector Control *In Vitro*	52350514
3	IACS-TRIM-20	MDA-MB-231	Breast Cancer Cell Line with TRIM28 Knockdown *In Vitro*	69233840
4	IACS-TRIM-21	MDA-MB-231	Breast Cancer Xenograft WT1	81200800
5	IACS-TRIM-22	MDA-MB-231	Breast Cancer Xenograft WT2	70977614
6	IACS-TRIM-23	MDA-MB-231	Breast Cancer Xenograft WT3	61127350
7	IACS-TRIM-24	MDA-MB-231	Breast Cancer Xenograft WT4	87621906
8	IACS-TRIM-25	MDA-MB-231	Breast Cancer Xenograft TRIM28 Knockdown 1	85849248
9	IACS-TRIM-26	MDA-MB-231	Breast Cancer Xenograft TRIM28 Knockdown 2	83705362
10	IACS-TRIM-27	MDA-MB-231	Breast Cancer Xenograft TRIM28 Knockdown 3	65253540

Supplementary Table S3: Gene sets downregulated in TRIM28^{KD} xenografts compared with control xenografts

No.	NAME OF GENE SETS	SIZE	ES	NES	NOM p-val	FDR q-val
1	SIGNALLING_BY_NGF	34	-0.338	**-2.349**	**0.002**	0.020
2	GPCR_DOWNSTREAM_SIGNALING	57	-0.251	**-2.234**	**0.002**	0.022
3	SIGNALING_BY_EGFR_IN_CANCER	19	-0.408	**-2.125**	**0.004**	0.035
4	SIGNALING_BY_GPCR	72	-0.202	**-2.010**	**0.008**	0.056
5	GASTRIN_CREB_SIGNALLING_PATHWAY_VIA_PKC_AND_MAPK	24	-0.329	**-1.888**	**0.019**	0.093
6	SIGNALING_BY_ERBB2	18	-0.368	**-1.872**	**0.004**	0.085
7	CLASS_A1_RHODOPSIN_LIKE_RECEPTORS	28	-0.295	**-1.859**	**0.011**	0.078
8	SIGNALING_BY_FGFR	21	-0.323	**-1.788**	**0.028**	0.098
9	DOWNSTREAM_SIGNALING_OF_ACTIVATED_FGFR	21	-0.323	**-1.776**	**0.014**	0.092
10	G_ALPHA_Q_SIGNALLING_EVENTS	21	-0.321	**-1.728**	**0.026**	0.105
11	SIGNALING_BY_FGFR_IN_DISEASE	23	-0.296	**-1.676**	**0.035**	0.124
12	PEPTIDE_LIGAND_BINDING_RECEPTORS	17	-0.344	**-1.670**	**0.029**	0.117
13	SIGNALING_BY_PDGF	20	-0.313	**-1.669**	**0.030**	0.109
14	GPCR_LIGAND_BINDING	41	-0.216	**-1.589**	**0.045**	0.148

ES, GSEA enrichment score; NES, GSEA normalized enrichment score; FDR, false discovery rate. Only gene sets that show $p < 0.05$ and FDR $q < 0.25$ are listed.

Supplementary Table S4: Gene sets upregulated in TRIM28^{KD} xenografts compared with control xenografts

No.	NAME OF GENE SETS	SIZE	ES	NES	NOM p-val	FDR q-val
1	SRP_DEPENDENT_COTRANSLATIONAL_ PROTEIN_TARGETING_TO_MEMBRANE	15	0.556	**2.611**	**0.000**	0.001
2	TRANSLATION	18	0.501	**2.523**	**0.000**	0.001
3	3_UTR_MEDIATED_TRANSLATIONAL_ REGULATION	16	0.493	**2.344**	**0.000**	0.003
4	INFLUENZA_LIFE_CYCLE	16	0.430	**2.102**	**0.004**	0.021
5	NONSENSE_MEDIATED_DECAY_ ENHANCED_BY_THE_EXON_JUNCTION_ COMPLEX	15	0.462	**2.091**	**0.000**	0.018
6	METABOLISM_OF_MRNA	29	0.288	**1.834**	**0.013**	0.079
7	MHC_CLASS_II_ANTIGEN_ PRESENTATION	18	0.357	**1.813**	**0.012**	0.075
8	METABOLISM_OF_PROTEINS	59	0.195	**1.732**	**0.012**	0.101
9	TRANSCRIPTIONAL_REGULATION_OF_ WHITE_ADIPOCYTE_DIFFERENTIATION	15	0.359	**1.664**	**0.036**	0.128

ES, GSEA enrichment score; NES, GSEA normalized enrichment score; FDR, false discovery rate. Only gene sets that show $p < 0.05$ and FDR $q < 0.25$ are listed.
