# Supplementary material for: TRIM28 multi-domain protein regulates cancer stem cell population in breast tumor development
Source: Oncotarget. 2016 Nov 10;8(1):863–82. doi: 10.18632/oncotarget.13273 (PMC5352203; doi:10.18632/oncotarget.13273)
Supplement: Supplementary file 2 [file oncotarget-08-863-s002.docx]

**Supplementary Table 1. Summary of TRIM28 differential expression analysis from Oncomine database.**

Gene expression comparison, Study authors and references, rank, percentile and differential expression statistics for TRIM28 overexpression is summarized.

| **COMPARISON** | **STUDY** | **GENE RANK** | **RANK (%)** | | **t-statistics** | | **FC** | **p-value** | **REFERENCE** |
| --- | --- | --- | --- | --- | --- | --- | --- | --- | --- |
| **Infiltrating Bladder Urothelial Carcinoma - Normal Bladder** | Dyrskjot | 111 | | **1** | | **6.875** | **2.813** | **1.82E-07** | Cancer Res 2004 |
| **Superficial bladder cancer - Normal Bladder** | Dyrskjot | 367 | | **3** | | **6.709** | **2.659** | **1.29E-07** | Cancer Res 2004 |
| **Superficial bladder cancer - Normal bladder** | Sanchez-Carbayo | 272 | | **3** | | **11.169** | **3.188** | **1.30E-17** | J Clin Oncol 2006 |
| **TCGA Glioblastoma – Brain** | TCGA | 739 | | **6** | | **13.196** | **1.695** | **4.75E-09** | TCGA GBM 2008 & 2013 |
| **Glioblastoma - Neural stem cell** | Lee | 464 | | **3** | | **-10.194** | **-2.114** | **3.40E-10** | Cancer Cell 2006 |
| **Invasive ductal breast carcinoma – breast** | Curtis | 763 | | **4** | | **24.913** | **1.588** | **1.39E-66** | Nature 2012 |
| **Medullary breast carcinoma - Normal breast** | Curtis | 1600 | | **9** | | **6.665** | **1.731** | **6.27E-08** | Nature 2012 |
| **Cervical Squamous Cell Carcinoma - Cervix squamous epithelium** | Scotto Cervix 2 | 750 | | **6** | | **4.666** | **1.558** | **1.04E-05** | Gene Chr Can 2008 |
| **Colon Adenoma – Normal** | Sabates-Bellver | 89 | | **1** | | **12.707** | **2.084** | **3.59E-18** | Mol Cancer Res 2007 |
| **Rectal Adenoma – Normal** | Sabates-Bellver | 1634 | | **9** | | **7.502** | **2.432** | **4.23E-05** | Mol Cancer Res 2007 |
| **Colon Adenocarcinoma – Colon** | Ki | 95 | | **2** | | **9.98** | **1.63** | **1.82E-16** | Int J Cancer 2007 |
| **Colorectal Adenocarcinoma - Colorectal tissue** | Skrzypczak | 167 | | **1** | | **10.413** | **1.55** | **9.10E-15** | PLOS ONE 2010 |
| **Colorectal carcinoma – Colon** | Hong | 605 | | **4** | | **11.785** | **2.627** | **2.35E-11** | Clin Exp Metastasis 2010 |
| **Colon Adenoma - Colon** | Skrzypczak 2 | 730 | | **4** | | **8.266** | **2.195** | **2.27E-06** | PLOS ONE 2010 |
| **Colon carcinoma – Colon** | Skrzypczak 2 | 965 | | **5** | | **10.925** | **2.23** | **8.08E-08** | PLOS ONE 2010 |
| **Rectal Adenomcarcinoma – Rectum** | Gaedcke | 1385 | | **8** | | **9.93** | **1.808** | **1.86E-17** | Gen Chr Canc 2010 |
| **Gastric Intestinal Adenocarcinoma - Gastric tissue** | Cho | 276 | | **2** | | **4.618** | **1.629** | **2.33E-05** | Clin Cancer Res 2011 |
| **Diffuse Gastric Adenocarcinoma - Gastric Tissue** | Cho | 371 | | **2** | | **5.208** | **1.686** | **2.20E-06** | Clin Cancer Res 2011 |
| **Gastric mixed Adenocarcinoma - Gastric Mucosa** | Derrico | 830 | | **5** | | **6.33** | **1.96** | **9.99E-05** | Eur J Cancer 2009 |
| **Head & Neck Cancer** | FriersonHF | 371 | | **5** | | **5.789** | **2.686** | **4.00E-05** | Am J Pathol 2002 |
| **T-Cell Acute Lymphoblastic Leukemia – PBMC** | Haferlach | 1121 | | **6** | | **12.596** | **1.793** | **6.58E-25** | J clin oncol 2010 |
| **Pro-B Acute Lymphoblastic Leukemia – PBMC** | Haferlach | 1894 | | **10** | | **8.218** | **1.541** | **5.98E-14** | J clin oncol 2010 |
| **Hepatocellular Carcinoma – Liver** | Rossler | 160 | | **2** | | **7.828** | **2.326** | **2.62E-09** | Cancer Res 2012 |
| **Hepatocellular Carcinoma – Liver** | Chen | 599 | | **6** | | **6.388** | **1.529** | **7.36E-10** | Mol Biol Cell 2002 |
| **Squamous cell lung carcinoma – normal** | Talbot | 46 | | **46** | | **9.113** | **1.969** | **3.53E-13** | Cancer Res 2005 |
| **Lung adenomcarcinoa – normal** | Beer | 48 | | **1** | | **8.011** | **1.527** | **8.44E-10** | Nat Med 2002 |
| **Lung adenocarcinoma – lung** | Selamat | 380 | | **2** | | **9.977** | **1.741** | **1.74E-16** | Genome Res 2012 |
| **Large cell Lung Carcinoma – Lung** | Hou | 983 | | **6** | | **5.962** | **2.108** | **3.46E-06** | PLOS ONE 2010 |
| **MGUS - Bone Marrow** | Zhan 3 | 1087 | | **6** | | **4.93** | **1.712** | **4.60E-06** | Blood 2007 |
| **Smoldering Myeloma** | Zhan 3 | 1308 | | **7** | | **6.547** | **2.829** | **1.36E-06** | Blood 2007 |
| **Testicular Seminoma – Testis** | Speger | 564 | | **6** | | **5.515** | **2.187** | **2.02E-06** | PNAS 2003 |
| **Myxoid/Round cell Liposarcoma - Adipose tissue** | Barretina Sarcoma | 809 | | **7** | | **7.845** | **1.993** | **4.33E-08** | Nat Genet 2010 |
| **Burkitt's lymphoma - B-lymphocyte** | Basso Lymphoma | 709 | | **9** | | **5.103** | **1.518** | **1.45E-05** | Nat Genet 2005 |
